# Piezocatalytic lithium titanate nanoparticles: a dual-action strategy against multidrug-resistant pathogens and cancer

**DOI:** 10.1039/d6ra05405f

**Published:** 2026-07-27

**Authors:** Karzan Qurbani, Haider Hamzah, Omid Amiri

**Affiliations:** a Department of Biology, College of Science, University of Raparin Rania City 46012 Kurdistan Region Iraq karzan.qurbani@uor.edu.krd; b Department of Biology, College of Science, University of Sulaimani Sulaymaniyah City 46001 Kurdistan Region Iraq; c Department of Medical Biochemical Analysis, Cihan University-Erbil Erbil City 44001 Kurdistan Region Iraq

## Abstract

Antimicrobial resistance (AMR) and colorectal cancer remain major global health challenges that necessitate the development of innovative therapeutic strategies. In this study, lithium titanate (Li_2_TiO_3_) nanoparticles were synthesized *via* a hydrothermal method and optimized as a multifunctional piezocatalytic nanoplatform for antimicrobial and anticancer applications. X-ray diffraction (XRD), scanning electron microscopy (SEM), transmission electron microscopy (TEM), energy-dispersive X-ray spectroscopy (EDX), Brunauer–Emmett–Teller (BET), and Fourier-transform infrared (FTIR) analyses confirmed the successful synthesis of highly crystalline monoclinic Li_2_TiO_3_ nanoparticles with a diamond-shaped morphology (25–80 nm), high purity, and physicochemical properties favorable for piezocatalytic activity. BET analysis further demonstrated that methylene blue (MB) removal was governed predominantly by piezocatalytic degradation rather than adsorption. Upon ultrasonic activation (300 W, 10 min), the optimized nanoparticles exhibited enhanced piezocatalytic performance, achieving 36.69% methylene blue degradation, accompanied by a 2.91-fold increase in singlet oxygen/superoxide (^1^O_2_/˙O_2_^−^) and a 2.39-fold increase in hydroxyl radical (˙OH) generation compared with non-sonicated nanoparticles. The enhanced reactive oxygen species (ROS) generation translated into potent antibacterial activity against both reference and drug-resistant pathogens, reducing the minimum inhibitory concentrations to 60 µg mL^−1^ for multidrug-resistant *Staphylococcus aureus* and 40 µg mL^−1^ for extensively drug-resistant *Pseudomonas aeruginosa*, while producing inhibition zones of 11.66 mm and 21.33 mm, respectively. The nanoparticles also exhibited remarkable antibiofilm activity, achieving up to 99.56% biofilm inhibition and 99.95% biofilm removal. Mechanistic investigations revealed that bacterial inactivation was mediated by ROS-induced plasma membrane depolarization and severe membrane disruption, as confirmed by DiBAC_4_(3) fluorescence analysis and transmission electron microscopy. Furthermore, the optimized nanoparticles demonstrated favorable hemocompatibility, minimal lithium-ion release, and significant cytotoxicity against HCT-116 colorectal cancer cells, with an IC_50_ of 100 µg mL^−1^ and an LC_50_ of 200 µg mL^−1^, inducing sustained morphological damage and growth inhibition for up to 96 h after treatment. Collectively, these findings establish sonication-activated Li_2_TiO_3_ nanoparticles as a biocompatible multifunctional piezocatalytic nanoplatform with considerable potential for combating multidrug-resistant bacterial infections and colorectal cancer through enhanced ROS-mediated therapeutic activity.

## Abstract 2

Antimicrobial resistance (AMR) and colorectal cancer remain major global health challenges that necessitate the development of innovative therapeutic strategies.

## Graphical abstract

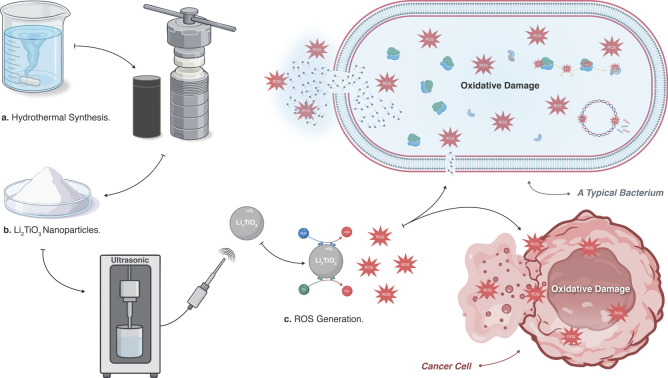

## 1. Introduction

Antimicrobial resistance (AMR) is an escalating global health threat that risks reversing decades of progress in infectious-disease control.^1,2^ Extensive use of broad-spectrum antibiotics, while clinically transformative, has accelerated the emergence of resistance and eroded the efficacy of key drug classes.^3,4^ Multidrug-resistant (MDR) pathogens now compromise first-line therapies, increasing mortality, length of stay, and healthcare costs.^5^ Particularly concerning is resistance to last-resort agents such as carbapenems and colistin in *Escherichia coli*, *Klebsiella pneumoniae*, and *Pseudomonas aeruginosa*.^6,7^ Current estimates attribute ≈700 000 deaths annually to AMR, potentially rising to 10 million by 2050 without effective countermeasures.^8,9^

AMR arises through a mosaic of mechanisms—spontaneous mutation, horizontal gene transfer, and adaptive responses to drug-imposed selection.^1,10^ Bacteria mitigate antibiotic action by producing drug-degrading enzymes, altering molecular targets, and activating multidrug efflux systems.^11^ Biofilm formation, a hallmark of chronic infection, further confers tolerance by embedding cells in a protective extracellular matrix that impedes antibiotic penetration and immune clearance.^12,13^ A high proportion of chronic microbial infections are biofilm-associated.^14^ The slow discovery pipeline for new antibiotics intensifies the need for alternative strategies that circumvent classical resistance routes.^15^

Non-antibiotic antimicrobials—including antimicrobial peptides, germicidal metal ions, and cationic polymers—offer complementary modes of action but are constrained by systemic toxicity and pharmacological limitations.^16,17^ Nanotechnology introduces further options: metal nanoparticles, quantum dots, and carbon-based platforms can disrupt membranes, interfere with metabolism, and generate reactive oxygen species (ROS),^18–20^ with high surface-area-to-volume ratios enabling potency at low doses.^21^ Yet unresolved questions around long-term biosafety, immunogenicity, and environmental persistence underscore the need for safer nano-therapeutics.^22^ Potential ecological risks from nanoparticle accumulation in soil and aquatic systems warrant careful consideration.^23^

Stimuli-responsive approaches enhance spatiotemporal control and can limit off-target toxicity. Photodynamic and photothermal modalities exemplify this principle but face hurdles including limited tissue penetration and collateral heating.^24,25^ Sonodynamic therapy (SDT) is a promising alternative: ultrasound-induced acoustic cavitation creates transient high-pressure/high-temperature microenvironments that catalyze ROS generation from sonosensitizers,^26,27^ enabling disruption of bacterial membranes and metabolic pathways.^28,29^ However, conventional sonosensitizers often suffer from rapid charge-carrier recombination and suboptimal pharmacokinetics, constraining ROS yield and translation. Clinical adoption of SDT for infectious indications remains limited to early-stage studies.^30^ Importantly, ROS-based nanotherapies have also shown promise in oncology, particularly for colorectal cancer, where oxidative stress induction and biofilm-like tumor microenvironments parallel challenges seen in bacterial resistance.^31,32^ Advances in materials science—defect engineering, surface functionalization, and hybrid nano-architectures—can improve charge separation and prolong ROS lifetimes.^33^ Piezoelectric nanomaterials are particularly attractive: mechanical perturbation generates internal electric fields that drive redox chemistry and ROS formation.^34^ Hydrothermal synthesis affords precise control of size, morphology, and crystallinity, thereby tuning piezocatalytic performance and enabling purposeful defect/surface engineering.^35,36^ Reports also describe hydrothermal routes yielding nanostructures with favorable biocompatibility profiles.^37^

Within this framework, the present study investigates hydrothermally synthesized Li_2_TiO_3_ nanoparticles (NPs) as a dual-functional piezocatalytic platform. The central hypothesis is that sonication-activated Li_2_TiO_3_ NPs generate ROS capable of producing potent antibacterial and antibiofilm effects against multidrug-resistant pathogens, while simultaneously inducing cytotoxic activity against colorectal cancer cells. The purpose of the research is to evaluate these dual biomedical functions within a coherent mechanistic context, thereby establishing Li_2_TiO_3_ NPs as a promising nanoplatform for addressing both antimicrobial resistance and colorectal cancer.

## 2. Material and method

### 2.1. Materials

Anatase titanium dioxide (TiO_2_) and lithium hydroxide (LiOH) (Merck, Germany) were used as precursor materials for the synthesis of Li_2_TiO_3_ nanoparticles. Hydrochloric acid (HCl) and sodium hydroxide (NaOH) (Scharlau, Spain) were used for pH adjustment, while absolute ethanol (100%; Chem-Lab NV, Belgium) and isopropanol (Merck, Germany) were used during sample preparation. Deionized water prepared in the laboratory was used throughout the study.

Microbiological media were obtained from Lab M (Neogen Europe Ltd., United Kingdom), whereas Luria–Bertani (LB) broth and agar were purchased from Merck (Germany). Dulbecco's Modified Eagle Medium (DMEM), fetal bovine serum (FBS), and trypsin–EDTA (0.25%) were purchased from Fisher Scientific (USA). *S. aureus* ATCC 6538, *P. aeruginosa* ATCC 9029, multidrug-resistant (MDR) *S. aureus*, extensively drug-resistant (XDR) *P. aeruginosa*, HCT-116 human colorectal cancer cells (ATCC CCL-247), and human dermal fibroblast (HDF) cells (ATCC PCS-201-012) were used in this study. Unless otherwise stated, all chemicals were of analytical grade and were used without further purification.

### 2.2. Synthesis of Li_2_TiO_3_ NPs

Lithium titanate (Li_2_TiO_3_) was synthesized *via* a hydrothermal method using anatase titanium dioxide (TiO_2_) and lithium hydroxide (LiOH) as precursors, both of analytical grade. In a typical synthesis, 0.08 mol of LiOH (3.3568 g) was dissolved in deionized water to prepare an aqueous solution, into which 0.04 mol of TiO_2_ (3.1912 g) was added. The total volume of the mixture was maintained at 40 mL, corresponding to 70% of the Teflon-lined hydrothermal autoclave's capacity. The suspension was stirred for 20 minutes to ensure homogeneity before being transferred to the autoclave.

To optimize the synthesis, individual parameters were varied while the others remained constant. Reaction durations of 16, 20, and 24 h, and temperatures of 150, 180, and 210 °C, were separately tested. Precursor concentrations were adjusted within the range of 0.054 mol LiOH (2.2658 g) with 0.028 mol TiO_2_ (2.2338 g) to 0.104 mol LiOH with 0.054 mol TiO_2_ in 40 mL of deionized water (Table S1). Autoclave fill ratios of 50% (30 mL), 70% (40 mL), and 90% (60 mL) were also evaluated. Each parameter was assessed independently to determine its effect on phase composition, morphology, and catalytic activity. Following hydrothermal treatment, the precipitate was collected by centrifugation, washed repeatedly with deionized water and absolute ethanol, and dried at 60 °C for 3 h in ambient air to yield a white Li_2_TiO_3_ powder, following a modified procedure from ref. 38.

### 2.3. Characterization of lithium titanate (Li_2_TiO_3_)

The structural, morphological, and functional properties of the synthesized Li_2_TiO_3_ nanoparticles were comprehensively characterized using a suite of advanced analytical techniques. Crystalline phases and structural integrity were initially assessed through X-ray diffraction (XRD; Philips X′Pert PRO, The Netherlands), confirming phase purity and the expected crystal structure. Elemental composition and spatial distribution of lithium, titanium, and oxygen were analyzed *via* energy-dispersive X-ray spectroscopy (EDS), with elemental mapping further verifying the uniform distribution of these elements across the samples. Surface morphology and particle size were examined using scanning electron microscopy (SEM; FEI Quanta 200, The Netherlands), providing detailed information on the nanoparticles' surface architecture. High-resolution transmission electron microscopy (TEM; JEOL JEM-2100 F, Japan) was employed to investigate the internal structure and crystal lattice spacing, offering deeper insights into the material's crystalline nature. The specific surface area and pore size distribution were quantified using Brunauer–Emmett–Teller (BET) analysis and Barrett–Joyner–Halenda (BJH) methods, respectively, *via* nitrogen adsorption–desorption isotherms (Micromeritics Tristar II 3020, USA). In addition, Fourier-transform infrared spectroscopy (FTIR) (Thermo Scientific Nicolet iS50, USA) was carried out in the range of 400–4000 cm^−1^ to confirm surface functional groups and lattice vibrations.

### 2.4. Piezocatalytic evaluation of Li_2_TiO_3_

The piezocatalytic degradation of methylene blue (MB) was employed as a model reaction to evaluate and optimize the catalytic performance of Li_2_TiO_3_ nanoparticles under ultrasonic stimulation.^39^ MB (10 µg mL^−1^) degradation was carried out under ultrasonication (300 W, 25 °C), and the decrease in absorbance at 664 nm was monitored using UV-Vis spectroscopy. A series of formulations (L1–L9) were initially screened based on MB removal efficiency, and the most active sample (L1) was selected for further optimization. Ultrasonic parameters, including irradiation time (1–60 min), applied power (0–500 W), and pulsed sonication modes, were systematically varied to maximize degradation efficiency. ROS generation was confirmed using selective chemical probes. 1,3-Diphenylisobenzofuran (DPBF) was used to detect singlet oxygen and superoxide species by monitoring the decrease in absorbance at 410 nm, while terephthalic acid fluorescence (Ex 315 nm) was employed for hydroxyl radical detection. These assays verified the formation of ROS under ultrasonic activation of Li_2_TiO_3_ nanoparticles.

Since ROS are the primary mediators of antimicrobial activity in piezocatalytic systems, the MB degradation assay and ROS analyses were used as preliminary optimization and mechanistic validation steps prior to antibacterial and antibiofilm evaluations.

Detailed protocols, reagent concentrations, optimization matrices, and raw spectra are provided in the SI (S1 and S2).

### 2.5. Active species identification

The role of ROS in the sonocatalytic degradation of methylene blue (MB) by Li_2_TiO_3_ nanoparticles was elucidated using a scavenger-trapping approach. For all experiments, L1 (Li_2_TiO_3_ subjected to sonication at 300 W for 10 min) was dispersed at a final concentration of 100 µg mL^−1^ in aqueous MB solutions (10 µg mL^−1^) prepared in deionized water.

Aliquots (10 mL) of the suspension were transferred into a quartz reactor and exposed to ultrasonic irradiation (300 W) under ambient conditions. To selectively quench individual reactive species, specific scavengers were introduced prior to sonication: isopropyl alcohol (IPA, 10 mM) as a hydroxyl radical (˙OH) scavenger, methanol (CH_3_OH, 10 mM) as a hole (h^+^) scavenger, and l-methionine (10 mM) as a superoxide anion (˙O_2_^−^) scavenger. A control experiment was conducted under identical conditions in the absence of scavengers. After 10 min of sonication, the suspensions were centrifuged at 8000 rpm for 10 min to separate the catalyst. The resulting supernatants were analyzed by UV-Vis spectrophotometry at *λ*_max_ = 664 nm to quantify residual MB concentrations. The degradation efficiency (*η*, %) was determined using the following equation:
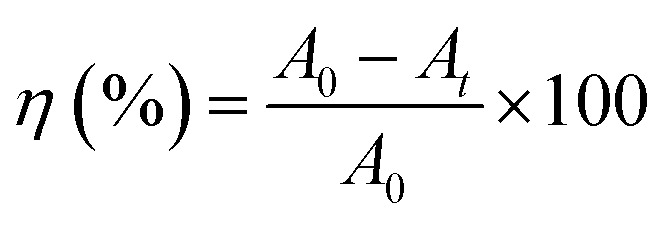
where: *A*_0_ = absorbance of MB solution at 664 nm before sonication (initial). *A*_*t*_ = absorbance of MB solution at 664 nm after sonication at time.

### 2.6. Antibacterial evaluation of Li_2_TiO_3_ nanoparticles

The antibacterial activity of Li_2_TiO_3_ nanoparticles was systematically evaluated against both standard strains (*Staphylococcus aureus* ATCC 6538 and *Pseudomonas aeruginosa* ATCC 9029) and resistant clinical isolates (MDR *S. aureus* and XDR *P. aeruginosa*). Nanoparticles synthesized under different hydrothermal conditions (L1–L9) were tested at a concentration range of 0, 1.25, 2.5, 5, 10, 20, 40, 60, 80, 100, 200, and 300 µg mL^−1^ in 96-well microtiter plates.^40^ Bacterial growth inhibition was monitored by measuring the optical density at 600 nm after incubation, allowing identification of the most effective formulations.

Antibacterial activity was further confirmed by a disc diffusion assay. Paper discs (6 mm, Whatman No. 1) were impregnated with 10 mg of Li_2_TiO_3_ nanoparticles prepared either as untreated powder or as aqueous suspensions subjected to ultrasonication (300 W, 10 min) prior to drying. To verify that the antibacterial activity originated from Li_2_TiO_3_ rather than residual TiO_2_, TiO_2_ nanoparticles were evaluated under identical conditions in both non-sonicated (T–O) and sonicated (T) forms. The discs were placed on Mueller–Hinton agar plates inoculated with bacterial suspensions standardized to 0.5 McFarland, followed by incubation at 37 °C for 24 h. Zones of inhibition were measured in millimetres.^41^ Azithromycin (15 mg per disc) served as the positive control.

The minimum inhibitory concentration (MIC) and minimum bactericidal concentration (MBC) were determined against all strains under both non-sonicated and sonicated conditions. Serial two-fold dilutions of nanoparticles (0, 1.25, 2.5, 5, 10, 20, 40, 60, 80, 100, 200, and 300 µg mL^−1^) were prepared in LB broth inoculated with 1 × 10^8^ CFU mL^−1^ bacterial suspensions. After 24 h incubation at 37 °C, MIC was defined as the lowest concentration that completely inhibited visible growth.^40^ For MBC determination, 5 µL aliquots from wells with no growth were spot-inoculated onto nutrient agar and incubated for a further 24 h at 37 °C; the MBC was recorded as the lowest concentration at which no colonies were observed, corresponding to a ≥99.9% reduction in viable bacteria.

### 2.7. Membrane potential assessment using bis-(1,3-dibutylbarbituric acid) trimethine oxonol (DiBAC_4_(3))

Bacterial membrane permeability changes were assessed using the membrane potential-sensitive fluorescent dye DiBAC_4_(3). For this analysis, bacterial strains (*S*.*aureus* ATCC strain and MDR clinical isolate; *P*. *aeruginosa* ATCC strain and XDR clinical isolate) were grown to mid-logarithmic phase in nutrient broth. Li_2_TiO_3_ nanoparticles (L1) were first sonicated at 300 W for 10 minutes to prepare the sonicated treatment group. Following growth, the bacterial suspensions (OD_600_ of 0.3, 2 mL) were mixed with DiBAC_4_(3) (200 µL, 5 µM) and incubated for 30 minutes at 37 °C. The mixtures were then divided into three treatment groups: PBS (control), non-sonicated Li_2_TiO_3_ nanoparticles (L1-O, 25 µg mL^−1^), and pre-sonicated Li_2_TiO_3_ nanoparticles (L1, 25 µg mL^−1^), with each treatment added to achieve a final volume of 4 mL. Membrane potential changes were monitored by measuring fluorescence intensity using a spectrofluorometer with excitation at 490 nm, where increased fluorescence indicated greater membrane permeability and depolarization.^39^

### 2.8. Biofilm production and antibiofilm activity of Li_2_TiO_3_ NPs

The biofilm production capacity of four bacterial strains—*S. aureus* (ATCC 6538 and MDR isolates) and *P. aeruginosa* (ATCC 9029 and XDR isolates)—was assessed to establish baseline biofilm-forming capabilities. The ATCC strains served as controls for comparison with the clinical isolates. Bacterial suspensions were prepared at an initial concentration of 1 × 10^6^ CFU mL^−1^ and inoculated into sterile 96-well microtiter plates containing Luria–Bertani (LB) broth. The plates were incubated at 37 °C for 48 hours under static conditions to allow biofilm development. Following the incubation period, the optical density (OD) at 600 nm was measured using a microplate reader to quantify biofilm biomass. LB broth without bacterial inoculation was included as a negative control to account for background absorbance. All experiments were conducted in triplicate, and the results were expressed as the mean OD ± standard deviation (SD).

The antibiofilm activity of Li_2_TiO_3_ nanoparticles (NPs) was evaluated using two complementary methods: biofilm reduction and biofilm removal. Sonicated Li_2_TiO_3_ nanoparticles (10 minutes at 300 W) were tested for biofilm reduction, while both sonicated and non-sonicated nanoparticles were evaluated for biofilm removal. Both *S. aureus* (ATCC strain and MDR clinical isolate) and *P. aeruginosa* (ATCC strain and XDR clinical isolate) were exposed to nanoparticles to assess their efficacy in reducing and removing biofilm formation.

For biofilm reduction, a modified MIC assay was employed. Bacterial suspensions of *S. aureus* (ATCC 6538 and MDR clinical isolate) and *P. aeruginosa* (ATCC 9029 and XDR clinical isolate) were prepared at a concentration of 1 × 10^8^ CFU mL^−1^ in LB broth. To each well of a 96-well microplate, 50 µL of bacterial suspension was added with 50 µL of Li_2_TiO_3_ nanoparticle suspensions at varying concentrations (0, 1.25, 2.5, 5, 10, 20, 40, 60, 80, 100, 200, and 300 µg mL^−1^). The final volume in each well was adjusted to 200 µL with LB broth. The plates were incubated at 37 °C for 48 hours to facilitate biofilm formation in the presence of nanoparticles. After incubation, non-adherent bacteria were removed by rinsing the wells gently with phosphate-buffered saline (PBS). Biofilms were stained with 200 µL of 0.1% crystal violet for 15 minutes at room temperature. Excess stain was washed off with PBS, and the crystal violet bound to biofilms was solubilized with 200 µL of 95% ethanol. Biofilm mass was quantified by measuring absorbance at 600 nm using a microplate reader.^42^

For biofilm removal, a modified crystal violet staining method was utilized. Biofilms were first allowed to form by inoculating 50 µL of bacterial suspension of *S. aureus* (ATCC 6538 and MDR clinical isolate) and *P. aeruginosa* (ATCC 9029 and XDR clinical isolate) into a 96-well microplate and incubating at 37 °C for 48 hours. After biofilm formation, 10 µL of Li_2_TiO_3_ nanoparticle suspensions at the same concentrations (0, 1.25, 2.5, 5, 10, 20, 40, 60, 80, 100, 200, and 300 µg mL^−1^) were added to the wells. The final volume was adjusted to 200 µL with LB broth, and both sonicated and non-sonicated nanoparticles were tested. The plates were incubated at room temperature for 2 hours to allow the nanoparticles to disrupt preformed biofilms. Following incubation, the wells were gently rinsed with PBS to remove any remaining planktonic bacteria. Residual biofilm was stained with 200 µL of 0.1% crystal violet for 15 minutes, followed by PBS washing. The bound crystal violet was solubilized with 200 µL of 95% ethanol, and absorbance at 600 nm was measured to quantify the biofilm mass.

### 2.9. Evaluation of memory effect on antibacterial activity

To investigate the memory effect of L1 on antibacterial activity, the minimum inhibitory concentration (MIC 50%) of L1 was determined and used for all four bacterial strains (*S. aureus* ATCC 6538, *S. aureus* MDR clinical isolate, *P*. *aeruginosa* ATCC 9029, and *P. aeruginosa* XDR clinical isolate). After ultrasonication at 300 W for 10 minutes, the L1 suspension was added to bacterial cultures at MIC 50% concentrations in nutrient broth. The mixtures were incubated at room temperature (25 °C), and at 1 hour, 24 hours, and 48 hours, aliquots were serially diluted and plated on nutrient agar. The colony-forming units per milliliter (CFU per mL) were counted to assess the remaining live cells. This analysis evaluated the sustained antibacterial effect of L1 over time.^28^

### 2.10. Cytotoxicity testing of Li_2_TiO_3_ NPs

The cytotoxicity of Li_2_TiO_3_ nanoparticles, specifically the sonicated formulation (L1) and non-sonicated formulation (L1-O), was evaluated on human dermal fibroblast (HDF) cells to determine their biocompatibility and impact on cell viability. HDF cells were cultured in Dulbecco's Modified Eagle Medium (DMEM) supplemented with 10% fetal bovine serum (FBS) and incubated at 37 °C in a humidified 5% CO_2_ atmosphere.

For the assay, 1 × 10^4^ cells were seeded into sterile 96-well tissue culture plates and incubated for 24 hours to allow for proper cell adhesion. Suspensions of Li_2_TiO_3_ nanoparticles were prepared at varying concentrations (0, 25, 50, 100, 200, 400, 600, 800, and 1000 µg mL^−1^). The sonicated formulation (L1) was obtained by subjecting the nanoparticle suspensions to ultrasonication at 300 W for 1 hour prior to cell exposure, while the non-sonicated formulation (L1-O) remained untreated.

Following 24 hours of exposure to the nanoparticle formulations, the culture medium was replaced with 100 µL of fresh DMEM containing 5 mg mL^−1^ MTT reagent. The cells were incubated for an additional 4 hours to allow viable cells to metabolize MTT into formazan crystals. Afterward, 100 µL of dimethyl sulfoxide (DMSO) was added to each well to dissolve the crystals, and the absorbance was measured at 490 nm using a microplate reader modified procedure from ref. 43.

### 2.11. *In vitro* hemocompatibility assessment

The hemotoxicity of Li_2_TiO_3_ nanoparticles was evaluated using human erythrocytes. Fresh blood was collected from a healthy volunteer (blood group A^+^) into citrate-stabilized tubes, following institutional ethical approval. Whole blood was centrifuged at 300 × *g* for 10 min to remove plasma, and erythrocytes were washed three times with phosphate-buffered saline (PBS, pH 7.4). A 2% erythrocyte suspension was prepared in PBS and used for the hemolysis assay.

Erythrocyte suspensions were exposed to either non-sonicated L1-O or sonicated L1, ultrasonicated at 300 W for 10 min, with azithromycin included as a reference drug control. Triton X-100 (Ferak Berlin GmbH, Germany) was used as a positive control to induce complete hemolysis, and PBS served as the negative control. Aliquots of 100 µL erythrocyte suspension were dispensed into 96-well plates and incubated with equal volumes of nanoparticle suspensions at concentrations of 0, 50, 100, 200, 400, 600, 800, and 1000 µg mL^−1^. Plates were incubated at 37 °C for 1 h. After incubation, the samples were centrifuged at 300 × *g* for 5 min, and 100 µL of the supernatant was transferred to fresh wells. Hemoglobin release was quantified by measuring absorbance at 540 nm using a microplate reader.

All experiments were performed in triplicate, and the percentage of hemolysis was calculated relative to Triton X-100 (100% lysis) and PBS (0% lysis) controls.^44^

The standard equation used in hemocompatibility studies to calculate hemolysis (%) is:
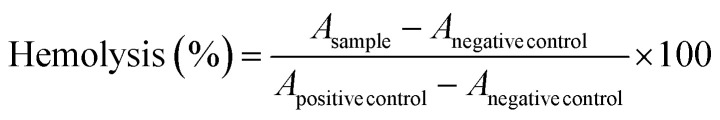
where: *A*_sample_ = absorbance of erythrocytes treated with nanoparticles or test compound (measured at 540 nm). *A*_negative control_ = absorbance of erythrocytes in PBS (baseline, 0% lysis). *A*_positive control_ = absorbance of erythrocytes treated with Triton X-100 (complete lysis, 100%).

### 2.12. Lithium-ion release study

Lithium-ion release from Li_2_TiO_3_ nanoparticles was quantified using inductively coupled plasma optical emission spectrometry (ICP-OES; Optima 2100 DV, PerkinElmer). Two nanoparticle formulations were assessed: L1-O (non-sonicated) and L1 (ultrasonicated at 300 W for 10 min). Suspensions of each formulation (1.0 mg mL^−1^; *n* = 3) were prepared in phosphate-buffered saline (PBS, pH 7.4) and Dulbecco's Modified Eagle Medium (DMEM) supplemented with 10% FBS. Samples were incubated at 37 °C under gentle agitation, and aliquots were collected at 1, 4, 24, 48, and 72 h. At each timepoint, aliquots were clarified by centrifugation (16 000 × *g*, 20 min, 4 °C) and filtration through 0.22 µm membranes, followed by stabilization of supernatants with 2% (v/v) HNO_3_. Total lithium content of the nanoparticles was determined separately *via* microwave-assisted acid digestion (HNO_3_/HCl, 5 : 1, v/v), permitting normalization of dissolved fractions. Quantification was performed using external multi-point calibration standards (0–10,000 µg L^−1^), and media blanks were included for background correction. Results are expressed as mean ± SD (*n* = 3) in µg L^−1^, and as cumulative release normalized to total lithium content (% release), following a modified protocol from Zhou *et al.* (2016).^45^

### 2.13. Anticancer activity of sonicated L1 NPs

The anticancer potential of L1 was investigated against HCT-116 colorectal cancer cells. Nanoparticle suspensions were pre-treated by ultrasonication (300 W, 1 h) to enhance dispersion and stability in the culture medium. Cytotoxicity was assessed by the MTT assay.^46^ Cells were seeded in 96-well plates at 1 × 10^4^ cells per well, incubated for 24 h at 37 °C in 5% CO_2_, and then treated for 24 h with graded concentrations of L1 (0, 25, 50, 100, 200, 400, 600, 800, and 1000 µg mL^−1^). Medium was replaced with 100 µL of DMEM containing MTT (5 mg mL^−1^) and incubated for 4 h; the resulting formazan crystals were dissolved in 100 µL DMSO, and absorbance was recorded at 570 nm (SpectraMax iD5, Molecular Devices). Cell viability was calculated relative to untreated controls, and IC_50_ and LC_50_ values were obtained by nonlinear regression (GraphPad Prism v9.0).

For mechanistic insights, membrane integrity was assessed by propidium iodide (PI) staining and flow cytometry.^47^ HCT-116 cells were seeded in 6-well plates at 5 × 10^5^ cells per well, exposed for 24 h to L1 at IC_50_ and LC_50_ concentrations, harvested with 0.25% trypsin–EDTA, centrifuged at 300×*g* for 5 min, and resuspended in complete medium containing 10% FBS. PI (5 µL, 50 µg mL^−1^) was added, and cells were incubated for 15 min in the dark before analysis with a BD FACSCalibur flow cytometer (FL-2 channel, 585/42 nm). A minimum of 10 000 events were collected per sample, and data were analyzed in FlowJo to quantify PI-positive cells.

Finally, morphological alterations were examined by scanning electron microscopy (SEM) following Ivask *et al.* (2018).^48^ Cells were cultured under two conditions—untreated controls and exposure to the LC_50_ concentration of L1 for 24 h—then fixed in 2.5% glutaraldehyde at 4 °C overnight, dehydrated in graded ethanol (10–100%), and subjected to critical-point drying. Dried samples were mounted on aluminium stubs, sputter-coated with gold, and imaged at multiple magnifications to assess membrane disruption, blebbing, shrinkage, and nanoparticle–cell interactions.

### 2.14. Memorial effect of sonicated L1 nanoparticles on cancer cells

To evaluate the long-term impact of sonicated L1 nanoparticles, the MTT assay was performed at multiple time points: 1 hour, 24 hours, 48 hours, 72 hours, and 96 hours, to assess any delayed cytotoxic effects and the persistence of nanoparticle activity. This approach allowed for the evaluation of potential “memorial” effects, where the influence of initial nanoparticle treatment might extend beyond the immediate exposure period. After each incubation period, the culture medium was replaced with 100 µL of fresh media containing 5 mg mL^−1^ MTT reagent, followed by a 4 hour incubation. Absorbance was measured at 570 nm, and viability data were normalized to untreated control cells. Nonlinear regression analysis in GraphPad Prism was employed to determine IC_50_ values at each time point, providing insights into the temporal dynamics of nanoparticle cytotoxicity and the sustained effects of sonication on HCT-116 cells (modified procedure from ref. 28).

### 2.15. Statistical analysis

All experiments were performed in triplicate unless otherwise stated, and results are expressed as mean ± standard deviation (SD). Pairwise comparisons between sonicated (L1) and non-sonicated (L1-O) nanoparticles were analyzed using independent-sample *t*-tests. Multi-factorial datasets, including piezocatalytic activity, antibacterial and antibiofilm assays, membrane potential changes, cytotoxicity, hemocompatibility, lithium-ion release, and anticancer evaluations, were analyzed by two-way analysis of variance (ANOVA) with Tukey's *post hoc* correction.

Normality and variance homogeneity were verified before applying parametric tests. Statistical significance was accepted at *p* < 0.05. All statistical analyses and data visualizations were performed using GraphPad Prism version 9.0.

### 2.16. Ethical statement

All procedures involving human participants were conducted in accordance with the ethical principles of the Declaration of Helsinki and approved by the Institutional Review Board (IRB) of the University of Raparin (Approval No. 75/2026). Informed consent was obtained from the human participant involved in this study.

## 3. Results

### 3.1. Characterization of Li_2_TiO_3_ NPs

#### 3.1.1 Optimization of hydrothermal synthesis parameters

**Fig. 1:**
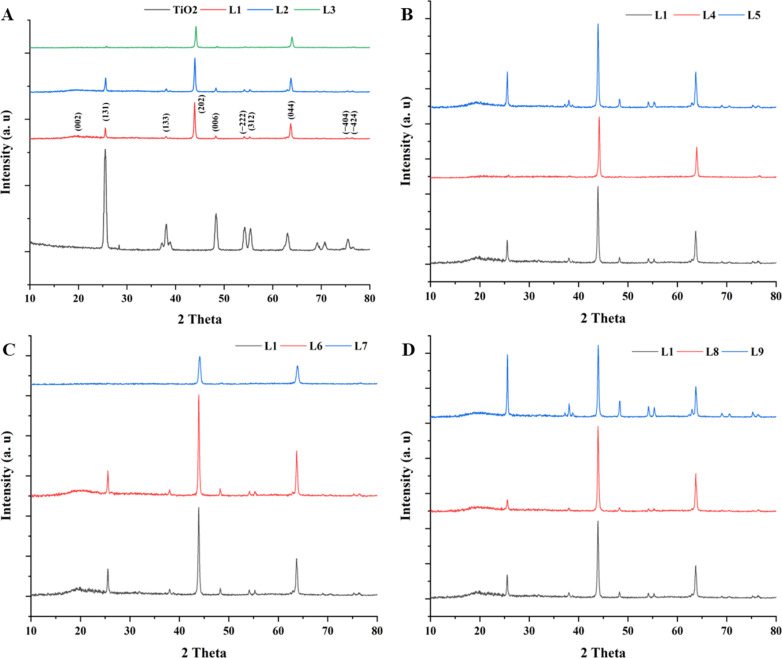
XRD patterns of L1 to L9. (A) L1–L3, (B) L1, L4, and L5, (C) L1, L6, and L7, (D) L1, L8, and L9. Reflections are indexed to monoclinic β-Li_2_TiO_3_ (JCPDS card no. 33-0831; space group *C*2/*c*).

**Fig. 2:**
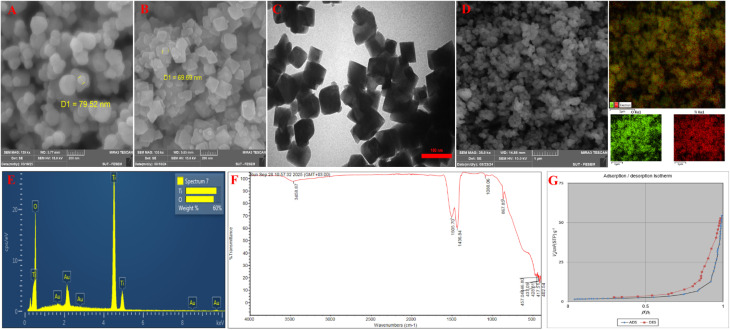
Physicochemical characterization of the optimized Li_2_TiO_3_ formulation (L1). (A) SEM micrograph of the TiO_2_ precursor, (B) SEM micrograph, (C) TEM micrograph, (D) SEM micrograph with corresponding elemental mapping, (E) EDX spectrum, (F) FTIR spectrum, and (G) BET surface area analysis.

A systematic investigation was conducted to optimize the hydrothermal synthesis parameters influencing Li_2_TiO_3_ formation, including reaction time, temperature, precursor concentration, and autoclave filling percentage. The synthesized products were characterized by X-ray diffraction (XRD) to determine their phase composition and crystallinity. The XRD pattern of the optimized sample (L1) is presented in Fig. 1A. Characteristic diffraction peaks were observed at 2*θ* = 19.38°, 25.55°, 38.03°, 43.91°, 48.28°, 54.09°, 55.28°, 63.66°, 75.30°, and 76.35°. These reflections were indexed to JCPDS card no. 33-0831, confirming the formation of monoclinic Li_2_TiO_3_ (space group *C*2/*c*). The strongest reflection at 43.91° corresponded to the (202) plane, while the remaining peaks were assigned to the (002), (133), (006), (−222), (312), (044), (−404), and (−424) planes, demonstrating excellent agreement with the reference pattern. A weak reflection at 25.55° was attributed to a trace secondary phase, such as anatase TiO_2_ or Li_2_CO_3_; however, its negligible intensity indicated minimal impurity. Broadening of the (002) reflection suggested nanoscale crystallite dimensions and possible lattice microstrain. Scherrer analysis estimated crystallite sizes of approximately 25–60 nm, which agreed well with the SEM (25–100 nm) and TEM (25–80 nm) observations (Fig. 2A–C; SI Fig. S1), confirming the formation of highly crystalline nanoscale Li_2_TiO_3_. The combination of high crystallinity, nanoscale dimensions, and minimal impurities provides favorable structural characteristics for enhanced piezocatalytic performance by facilitating efficient charge separation and transport while exposing catalytically active surface facets.

##### 3.1.1.1 Effect of hydrothermal synthesis parameters

Hydrothermal synthesis parameters markedly influenced the phase composition and piezocatalytic performance of Li_2_TiO_3_ (Fig. 1A–D). Reaction time governed phase evolution, with pure Li_2_TiO_3_ obtained after 16 h, formation of a TiO_2_/Li_2_TiO_3_ composite after 20 h, and Li_2_TiO_3_ becoming the dominant phase after 24 h, which exhibited the highest piezocatalytic activity (Fig. 1A). Likewise, increasing the hydrothermal temperature from 150 to 210 °C progressively promoted Li_2_TiO_3_ formation, whereas the TiO_2_/Li_2_TiO_3_ composite obtained at 180 °C exhibited enhanced piezocatalytic activity, indicating a synergistic interaction between the two phases (Fig. 1B). LiOH concentration also affected phase composition, with TiO_2_/Li_2_TiO_3_ composites formed at 0.05 and 0.08 mol, whereas pure Li_2_TiO_3_ was obtained at 0.10 mol (Fig. 1C), highlighting the importance of precursor stoichiometry. Similarly, the autoclave filling percentage influenced crystal formation; Li_2_TiO_3_ remained the dominant phase at 50% and 70% filling, whereas TiO_2_ predominated at 90%, likely owing to changes in hydrothermal pressure and reagent diffusion within the reaction vessel (Fig. 1D).

Collectively, these results demonstrate that precise control of hydrothermal synthesis parameters enables systematic regulation of phase composition and crystallinity, thereby providing an effective strategy for optimizing the piezocatalytic performance of Li_2_TiO_3_.

#### 3.1.2 Morphological and structural analysis *via* SEM

SEM was employed to evaluate the influence of hydrothermal synthesis parameters on the morphology of Li_2_TiO_3_ (Fig. 2B; SI Fig. S1). Hydrothermal reaction time markedly affected particle size. After 16 h, most particles exceeded 100 nm in diameter, whereas extending the reaction time to 20 and 24 h progressively reduced particle size. This reduction coincided with the transition from Li_2_TiO_3_ to a TiO_2_/Li_2_TiO_3_ composite, indicating a close relationship between phase evolution and crystal growth.

Hydrothermal temperature also influenced morphology and aggregation. At 150 °C, highly aggregated and irregular morphologies were observed. Increasing the temperature to 180 °C reduced aggregation and produced more uniformly distributed particles, whereas synthesis at 210 °C yielded pure Li_2_TiO_3_ with a homogeneous morphology, indicating improved crystallization (SI Fig. S1).

Precursor concentration further affected morphology. Samples L6 and L1 exhibited comparable particle size and surface morphology, consistent with their similar phase composition identified by XRD. In contrast, sample L7, composed predominantly of Li_2_TiO_3_, exhibited smaller particles, demonstrating that precursor stoichiometry contributed to controlling particle growth under the selected synthesis conditions (SI Fig. S1). In contrast, varying the autoclave filling percentage produced no appreciable changes in particle size or morphology, although it influenced the phase composition of the synthesized products. Overall, hydrothermal reaction time, temperature, and precursor concentration governed the morphological evolution of Li_2_TiO_3_, whereas autoclave filling percentage had only a limited influence on particle morphology.

#### 3.1.3 Elemental composition analysis *via* EDX

Energy-dispersive X-ray spectroscopy (EDX) was performed to confirm the elemental composition of the synthesized materials. The results indicated the presence of oxygen (O), titanium (Ti), and gold (Au) in all samples (Fig. 2D and E) (SI Fig. S2 and S3). The absence of a detectable lithium (Li) peak can be attributed to its low atomic number, which limits its detectability *via* EDX. The Au peak originated from the conductive coating applied during SEM sample preparation. These findings confirm the high purity of the synthesized materials, with no detectable impurities.

#### 3.1.4 Structural and morphological characterization *via* TEM

Transmission electron microscopy (TEM) analysis was conducted on the optimal sample, L1, to further examine its morphology and structural properties. The results revealed a well-defined diamond-shaped morphology, with particle sizes ranging between 25 and 80 nm (Fig. 2C). TEM imaging provided additional confirmation of the refined structural characteristics observed in SEM analysis, reinforcing the consistency and reliability of the synthesis approach.

#### 3.1.5 Surface area analysis of methylene blue removal by Li_2_TiO_3_ NPs

To discern whether the removal of methylene blue (MB) was primarily driven by adsorption or piezocatalytic activity, BET surface area analysis was performed on the two most efficient samples (L1 and L2) and the two least efficient samples (L5 and L7). The results were striking: L1, exhibiting the highest piezocatalytic activity, demonstrated the lowest surface area, whereas L7, which exhibited the lowest piezocatalytic performance, had the highest surface area (Fig. 2G) and (SI Fig. S4A–C). These findings suggest a strong inverse correlation between surface area and piezocatalytic efficiency, thereby providing compelling evidence that MB removal is primarily attributed to piezocatalytic degradation rather than adsorption.

#### 3.1.6 FTIR spectroscopy

FTIR spectroscopy was conducted to confirm the bonding environment and structural features of the hydrothermally synthesized Li_2_TiO_3_ nanoparticles (Fig. 2F and S9). A broad absorption centered at 3459 cm^−1^ corresponds to O–H stretching vibrations from surface hydroxyl groups and adsorbed water. Distinct bands at 1506, 1437, 1088, and 868 cm^−1^ are assigned to carbonate (CO_3_^2−^) stretching and bending modes, which most likely arise from atmospheric CO_2_ adsorption on the titanate surface during post-synthesis handling. In the low-wavenumber region, strong absorptions between 457 and 403 cm^−1^ are attributed to Ti–O and Li–O lattice vibrations, providing spectroscopic confirmation of the crystalline Li_2_TiO_3_ framework in agreement with XRD results.

### 3.2. Optimization of Li_2_TiO_3_ NPs for piezocatalytic and antibacterial applications

The optimization of Li_2_TiO_3_ nanoparticles (NPs) was conducted to enhance their piezocatalytic activity for methylene blue (MB) degradation and to evaluate their potential for antibacterial applications. This systematic study assessed various formulations (L1-L9) to identify the optimal synthesis parameters.

#### 3.2.1 MB removal efficiency of Li_2_TiO_3_ formulations (L1–L9)

**Fig. 3:**
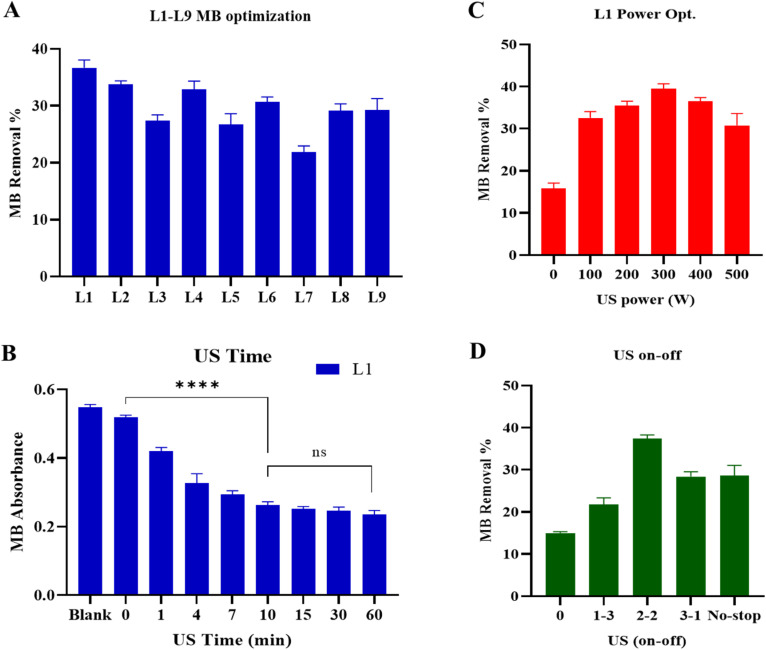
Optimization of Li_2_TiO_3_ NPs based on methylene blue removal. (A) MB removal efficiency of Li_2_TiO_3_ formulations (L1–L9), highlighting the superior performance of L1, (B) sonication time optimization for MB degradation, (C) effect of ultrasonication power on MB removal efficiency, (D) impact of on–off ultrasonication cycles on MB removal.

The piezocatalytic efficiency of the Li_2_TiO_3_ formulations was evaluated by mixing a 10 µg mL^−1^ MB solution with 100 µg mL^−1^ of each formulation, followed by 10 minutes of ultrasonication at 300 W. Among the tested formulations, L1 demonstrated the highest MB removal efficiency, achieving a degradation rate of 36.69%. L2 followed with 33.79%, while L7 exhibited the lowest efficiency at 21.86% (Fig. 3A). These results underscore the superior catalytic properties of L1, establishing it as the optimal formulation for further studies.

#### 3.2.2 Sonication time optimization for MB degradation

The optimal formulation, L1 (100 µg mL^−1^), was evaluated under varying sonication times (0–60 min) to determine the ideal duration for MB degradation. A progressive decrease in MB absorbance was observed, with a marked reduction at 10 min from 0.518 to 0.263, corresponding to maximum degradation efficiency. Prolonged sonication up to 60 min did not produce significant additional decreases, with a final absorbance of 0.236 recorded at 60 min (Fig. 3B).

In contrast, the reference blank (MB solution without nanoparticles) exhibited minimal change under identical ultrasonic conditions, with absorbance decreasing only from 0.556 at 0 min to 0.532 at 60 min (SI Fig. S5), indicating negligible degradation. These results confirm that the observed efficiency is attributable to nanoparticle-mediated piezocatalysis rather than ultrasound alone.

#### 3.2.3 Effect of ultrasonication power on MB removal efficiency

The influence of ultrasonication power on MB removal was examined at power levels of 0, 100, 200, 300, 400, and 500 W. The results revealed that 300 W was the optimal power setting, achieving an MB removal efficiency of 39.42%. Beyond this level, a decline in activity was observed, with efficiency decreasing to 30.75% at 500 W. At 0 W, the removal efficiency was limited to 15.78%, indicating minimal catalytic activity in the absence of ultrasonication (Fig. 3C). These results highlight the critical role of precise power optimization in maximizing the piezocatalytic performance of Li_2_TiO_3_ NPs.

#### 3.2.4 Impact of on–off ultrasonication cycles on MB removal

To further optimize the activation conditions, the effect of on–off ultrasonication cycles on MB removal was evaluated. Four cycling methods were tested: continuous ultrasonication (0 seconds off), no stop, 1–3 seconds on–off, 2–2 seconds on–off, and 3–1 seconds on–off. The 2–2 seconds on–off cycle emerged as the most effective, achieving an MB removal efficiency of 37.38%. In comparison, the 1–3 seconds on–off cycle achieved 21.8% efficiency, and the 3–1 seconds on–off cycle reached 28.4%. In the absence of ultrasonication (0), the efficiency was minimal at 15.02%. These findings highlight the superiority of the 2–2 seconds on–off cycle in activating the piezocatalytic potential of Li_2_TiO_3_ NPs (Fig. 3D).

### 3.3. ROS generation by Li_2_TiO_3_ NPs

ROS, including singlet oxygen/superoxide species (^1^O_2_/˙O_2_^−^) and hydroxyl radicals (˙OH), were quantified to evaluate the piezocatalytic activity of Li_2_TiO_3_ nanoparticles synthesized under different conditions. Sonicated L1 nanoparticles generated substantially higher ROS levels than their non-sonicated counterpart (L1-O), with ^1^O_2_/˙O_2_^−^ and ˙OH production increasing by 2.91-fold and 2.39-fold, respectively, demonstrating the pronounced enhancement in piezocatalytic activity induced by sonication (SI Fig. S6A).

To elucidate the contribution of individual ROS species to methylene blue (MB) degradation, radical-scavenging experiments were performed using sonicated L1 under ultrasonic irradiation. In the absence of scavengers, L1 achieved 38.35% MB degradation after 10 min. The addition of isopropyl alcohol (IPA), a hydroxyl radical (˙OH) scavenger, reduced degradation efficiency to 18.22%, confirming that ˙OH was the predominant reactive species. Likewise, methanol (CH_3_OH), used as a hole (h^+^) scavenger, and l-methionine, employed to quench ˙O_2_^−^, reduced degradation efficiencies to 21.70% and 28.83%, respectively (SI Fig. S7), indicating secondary contributions from photogenerated holes and superoxide radicals. Comparison of all synthesized formulations (L1, L2, L5, and L7) and their corresponding non-sonicated counterparts demonstrated that L1 generated the highest ROS levels (SI Fig. S6B), confirming it as the optimum formulation for piezocatalytic applications.

Overall, these results demonstrate that sonication significantly enhances the piezocatalytic activity of Li_2_TiO_3_ by promoting ROS generation, with hydroxyl radicals serving as the principal oxidizing species, while photogenerated holes and superoxide radicals contribute synergistically to MB degradation.

### 3.4. Antibacterial activity of Li_2_TiO_3_ NPs

The antibacterial activity of Li_2_TiO_3_ nanoparticles synthesized under different conditions (L1–L9) was evaluated against *S. aureus* (ATCC 6538 and MDR clinical isolate) and *P. aeruginosa* (ATCC 9029 and XDR clinical isolate) over a concentration range of 1.25–300 µg mL^−1^. Among all formulations, L1 exhibited the strongest antibacterial activity, completely inhibiting MDR *S. aureus* and XDR *P. aeruginosa* at 100 and 80 µg mL^−1^, respectively. In contrast, the remaining formulations (L2–L9) required 300 µg mL^−1^ to achieve complete inhibition of both resistant isolates (SI Fig. S8A and B), identifying L1 as the optimum formulation for subsequent antibacterial investigations.

**Fig. 4:**
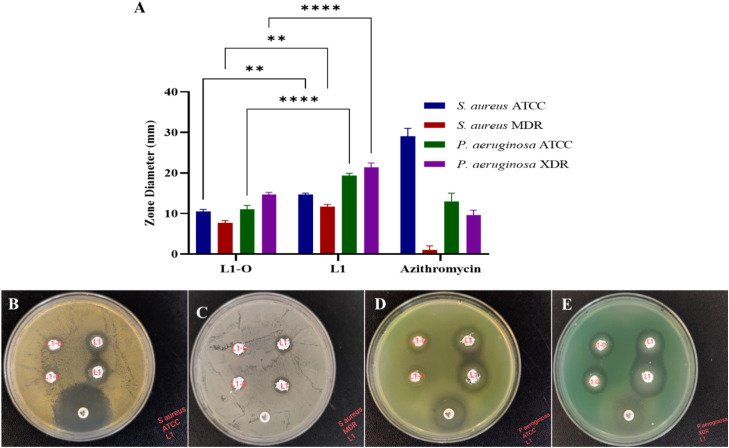
Antibacterial activity of Li_2_TiO_3_ NPs against drug-resistant pathogens. (A) Quantitative comparison of inhibition zones for sonicated and non-sonicated nanoparticles *versus* azithromycin control. (B–E) Representative disc diffusion assays on Mueller–Hinton agar showing zones of inhibition against *S. aureus* ATCC (B), *S. aureus* MDR (C), *P. aeruginosa* ATCC (D), and *P. aeruginosa* XDR (E).

The superior antibacterial performance of L1 was further confirmed using the Kirby–Bauer disc diffusion assay (Fig. 4). Sonicated L1 (300 W, 10 min) produced substantially larger inhibition zones than the non-sonicated formulation (L1-O). Against *P. aeruginosa*, the inhibition zones increased from 11.00 ± 0.80 to 19.33 ± 0.47 mm for the ATCC strain and from 14.66 ± 0.47 to 21.33 ± 0.94 mm for the XDR isolate. Likewise, inhibition zones against *S. aureus* increased from 10.50 ± 0.40 to 14.75 ± 0.20 mm for the ATCC strain and from 7.66 ± 0.50 to 11.66 ± 0.47 mm for the MDR isolate. By comparison, azithromycin (15 µg per disc) produced inhibition zones of 30 mm against *S. aureus* ATCC, 9 mm against the MDR isolate, 12 mm against *P. aeruginosa* ATCC, and 8 mm against the XDR isolate. In contrast, neither non-sonicated TiO_2_ (T–O) nor sonicated TiO_2_ (T) produced detectable inhibition zones against MDR *S. aureus* or XDR *P. aeruginosa* under identical experimental conditions (SI Fig. S9). These findings demonstrate that ultrasonic activation of TiO_2_ alone was insufficient to produce antibacterial activity, confirming that the antibacterial activity observed in the present study is attributable to Li_2_TiO_3_ rather than residual TiO_2_.

**Fig. 5:**
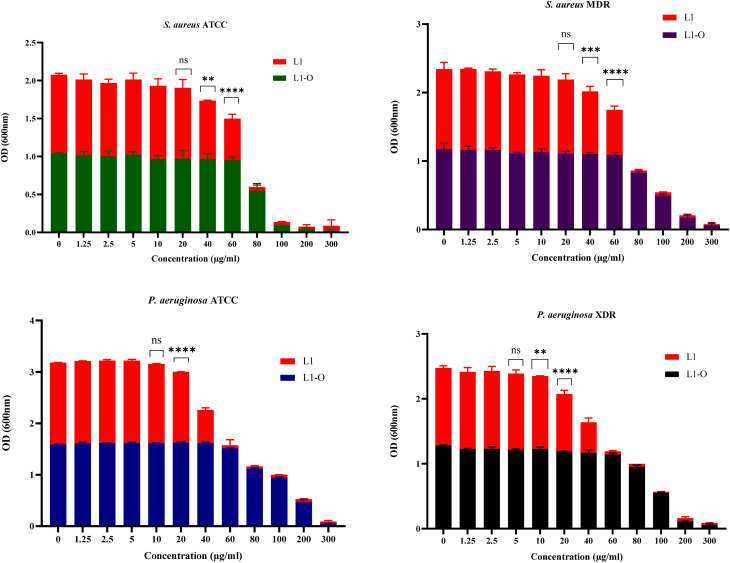
MIC and MBC of Li_2_TiO_3_ nanoparticles against *S. aureus* (ATCC 6538 reference strain and MDR clinical isolate) and *P. aeruginosa* (ATCC 9029 reference strain and XDR clinical isolate). MIC and MBC values were determined for both non-sonicated (L1-O) and sonicated (L1) formulations.

MIC and MBC analyses further confirmed the enhanced antibacterial efficacy of sonicated L1. For *S. aureus* ATCC, non-sonicated Li_2_TiO_3_ nanoparticles exhibited a MIC of 100 µg mL^−1^ and an MBC of 300 µg mL^−1^, whereas sonicated nanoparticles significantly reduced these values to a MIC of 60 µg mL^−1^ and an MBC of 80 µg mL^−1^. Similarly, in *S. aureus* MDR strains, the MIC and MBC for non-sonicated nanoparticles were 100 µg mL^−1^ and 200 µg mL^−1^, respectively. After sonication, the MIC decreased to 60 µg mL^−1^ and the MBC to 80 µg mL^−1^, demonstrating the enhanced efficacy of the sonicated formulation (Fig. 5 and SI Fig. S10).

For *P. aeruginosa* ATCC, non-sonicated nanoparticles required a MIC of 200 µg mL^−1^ to inhibit bacterial growth, while sonicated nanoparticles achieved the same effect at 40 µg mL^−1^, with an MBC of 60 µg mL^−1^. In the case of *P. aeruginosa* XDR, non-sonicated nanoparticles exhibited a MIC of 100 µg mL^−1^ and an MBC of 200 µg mL^−1^. Sonication further enhanced their efficacy, reducing the MIC to 40 µg mL^−1^ and the MBC to 60 µg mL^−1^ (Fig. 5 and SI Fig. S9).

These results underscore the significant improvements in antibacterial performance achieved through sonication, likely due to enhanced catalytic activity and increased surface interactions. The ability of sonicated Li_2_TiO_3_ nanoparticles to inhibit bacterial growth at lower concentrations highlights their potential as effective antimicrobial agents, particularly against multidrug-resistant pathogens.

### 3.5. Antibiofilm activity of Li_2_TiO_3_ NPs

The biofilm-forming capacity of *S. aureus* (ATCC 6538 and MDR clinical isolate) and *P. aeruginosa* (ATCC 9029 and XDR clinical isolate) was evaluated before assessing the antibiofilm activity of Li_2_TiO_3_ nanoparticles. Among the tested strains, *S. aureus* ATCC exhibited the highest biofilm production (OD_600_ = 1.282 ± 0.07), followed by *P. aeruginosa* ATCC (0.945 ± 0.05), *P. aeruginosa* XDR (0.766 ± 0.03), and *S. aureus* MDR (0.605 ± 0.013) (SI Fig. S11), demonstrating strain-dependent differences in biofilm-forming capacity.

**Fig. 6:**
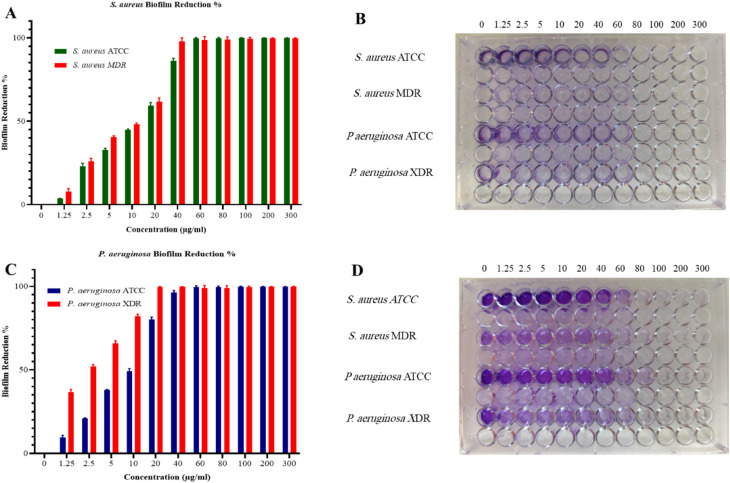
Biofilm reduction of Li_2_TiO_3_ NPs. (A) Percentage reduction in biofilm mass of *S. aureus* ATCC 6538, *S. aureus* MDR after treatment with sonicated Li_2_TiO_3_ nanoparticles at varying concentrations. (B) Percentage reduction in biofilm mass of *P. aeruginosa* ATCC 9029, and *P. aeruginosa* XDR after treatment with sonicated Li_2_TiO_3_ nanoparticles at varying concentrations. (C) Image of a 96-well microplate stained with crystal violet, illustrating biofilm formation before ethanol suspension. (D) Image of the same plate post-ethanol suspension, showing the biofilm mass after solubilization of the crystal violet.

The biofilm reduction activity of sonicated Li_2_TiO_3_ nanoparticles was subsequently evaluated against the four bacterial strains. At 20 µg mL^−1^, biofilm reduction reached 59.29% for *S. aureus* ATCC, 60.60% for *S. aureus* MDR, 80.07% for *P. aeruginosa* ATCC, and 99.56% for *P. aeruginosa* XDR. Biofilm reduction exceeding 99% was achieved at 20 µg mL^−1^ for *P. aeruginosa* XDR, 40 µg mL^−1^ for *S. aureus* MDR, and 60 µg mL^−1^ for both *S. aureus* ATCC and *P. aeruginosa* ATCC (Fig. 6).

**Fig. 7:**
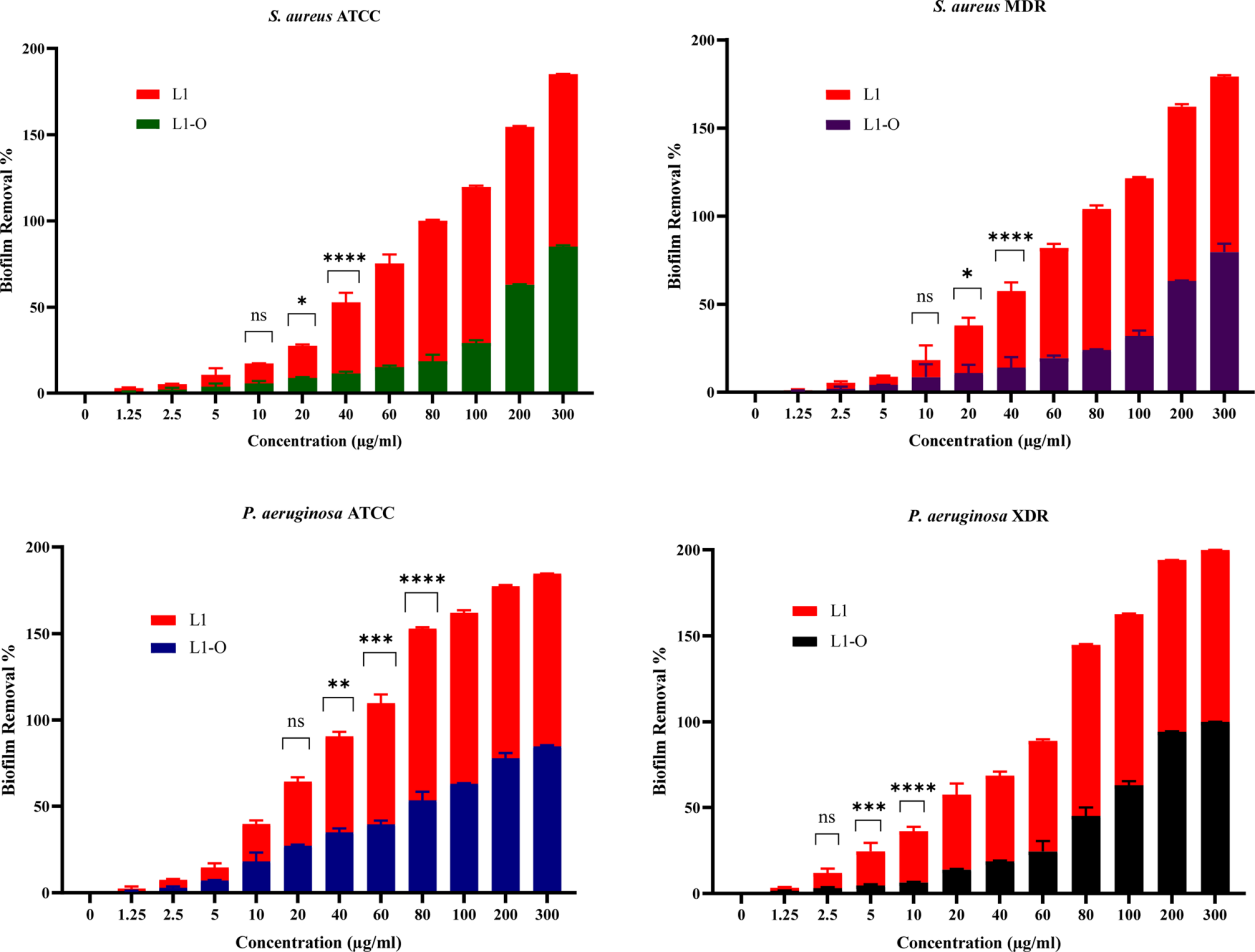
Biofilm removal activity of Li_2_TiO_3_ nanoparticles against bacterial strains. Removal percentage of biofilm formed by Gram positive *S. aureus* (ATCC strain and MDR clinical isolate) and Gram-negative *P. aeruginosa* (ATCC strain and XDR clinical isolate) treated with sonicated (L1) and non-sonicated (L1-O) Li_2_TiO_3_ nanoparticles.

Biofilm removal assays further demonstrated the superior performance of sonicated Li_2_TiO_3_ nanoparticles compared with the non-sonicated formulation (Fig. 7). For *S. aureus* ATCC and MDR, 300 µg mL^−1^ sonicated nanoparticles achieved 99.95% and 99.50% biofilm removal, respectively, compared with 85.20% and 79.59% for the corresponding non-sonicated nanoparticles. Likewise, sonicated nanoparticles achieved 99.45% and 99.63% biofilm removal against *P. aeruginosa* ATCC and XDR, respectively, at only 80 µg mL^−1^, whereas the non-sonicated formulation removed only 53.41% and 45.10% of the corresponding biofilms under the same conditions.

Overall, ultrasonic activation markedly enhanced both the biofilm reduction and biofilm removal activities of Li_2_TiO_3_ nanoparticles, confirming their excellent antibiofilm efficacy against both reference and multidrug-resistant bacterial strains.

### 3.6. Mechanism of action of Li_2_TiO_3_ NPs against pathogenic bacteria

**Fig. 8:**
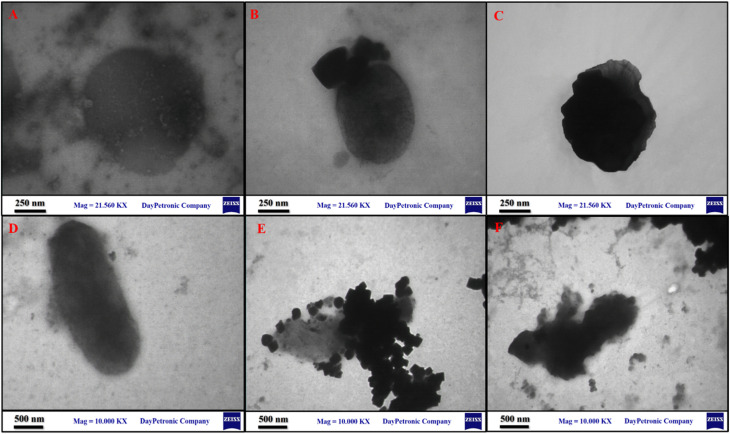
TEM images showing the effects of Li_2_TiO_3_ NPs piezocatalyst on bacterial cells. (A) Untreated *S*.*aureus* MDR cells with intact morphology. (B) *S. aureus* MDR cells with Li_2_TiO_3_ NPs attached to their surface. (C) Damaged *S. aureus* MDR cells after treatment with Li_2_TiO_3_ NPs. (D) Untreated *P*. *aeruginosa* XDR cells. (E and F) *P. aeruginosa* XDR cells treated with Li_2_TiO_3_ NPs, showing membrane disruption and cytoplasmic leakage.

TEM was employed to investigate the interaction of Li_2_TiO_3_ NPs with multidrug-resistant *S. aureus* (MDR) and extensively drug-resistant *P. aeruginosa* (XDR). Compared with the untreated controls, both bacterial species exhibited pronounced ultrastructural alterations following nanoparticle treatment (Fig. 8). In *S. aureus* MDR, Li_2_TiO_3_ NPs adhered to the bacterial surface, resulting in deformation of the cell envelope and localized membrane disruption (Fig. 8A–C). Although membrane disruption was evident, the overall cellular architecture remained largely preserved. By contrast, *P. aeruginosa* XDR exhibited substantially greater structural damage than the untreated control (Fig. 8D). TEM images revealed extensive membrane disruption, leakage of intracellular contents, and collapse of bacterial cells following nanoparticle attachment (Fig. 8E and F). Overall, TEM analysis confirmed that treatment with Li_2_TiO_3_ nanoparticles induced membrane disruption and loss of cellular integrity in both bacterial species, with more extensive ultrastructural damage observed in *P. aeruginosa* XDR than in *S. aureus* MDR (Fig. 8).

### 3.7. Plasma membrane integrity assessment using DiBAC_4_(3)

The effect of Li_2_TiO_3_ nanoparticles on plasma membrane permeability was evaluated using the fluorescent probe DiBAC_4_(3) for *S*.*aureus* (ATCC strain and MDR clinical isolate) and *P*. *aeruginosa* (ATCC strain and XDR clinical isolate) strains. Bacterial cells were treated with buffer (PBS), sonicated Li_2_TiO_3_ nanoparticles (L1), and non-sonicated Li_2_TiO_3_ nanoparticles (L1-O), and their membrane integrity was assessed.

The results demonstrated that non-sonicated nanoparticles (L1-O) disrupted bacterial plasma membranes to some extent, increasing permeability compared to the PBS control. However, sonicated nanoparticles (L1) significantly enhanced this effect, showing a dramatic increase in plasma membrane disruption. The impact was particularly pronounced in *P. aeruginosa* (ATCC strain and XDR clinical isolate), reflecting the heightened susceptibility of Gram-negative bacteria to nanoparticle-induced damage (SI Fig. S12). In *S. aureus* (ATCC strain and MDR clinical isolate), while both sonicated and non-sonicated nanoparticles increased permeability, the effect of sonicated nanoparticles was notably stronger. These observations align with the structural differences between Gram-positive and Gram-negative bacteria, where the thinner outer membrane of Gram-negative strains makes them more vulnerable to nanoparticle interaction (SI Fig. S12). These findings confirm the ability of Li_2_TiO_3_ nanoparticles to disrupt bacterial plasma membranes, with sonication significantly amplifying their antibacterial efficacy, particularly against Gram-negative pathogens.

### 3.8. Evaluation of memory effect of piezocatalytic activity

The memory effect of the piezocatalytic activity of L1 nanoparticles was assessed by monitoring MB removal over time after the initial 10-minute sonication. The degradation of MB was measured at 1-hour, 24-hour, 48-hour, 72-hour, and 96-hour intervals without additional sonication. The results demonstrated a gradual increase in MB removal over time. At 1 hour, MB removal was 35%, which increased to 59.07% after 24 hours, and reached 93.36% after 120 hours (SI Fig. S13). These results suggest that the Li_2_TiO_3_ nanoparticles exhibit a strong memory effect, retaining their piezocatalytic activity over extended periods, even without further sonication, indicating their long-term stability and sustained catalytic performance.

### 3.9. Memory effect of sonicated Li_2_TiO_3_ NPs on antibacterial activity

**Fig. 9:**
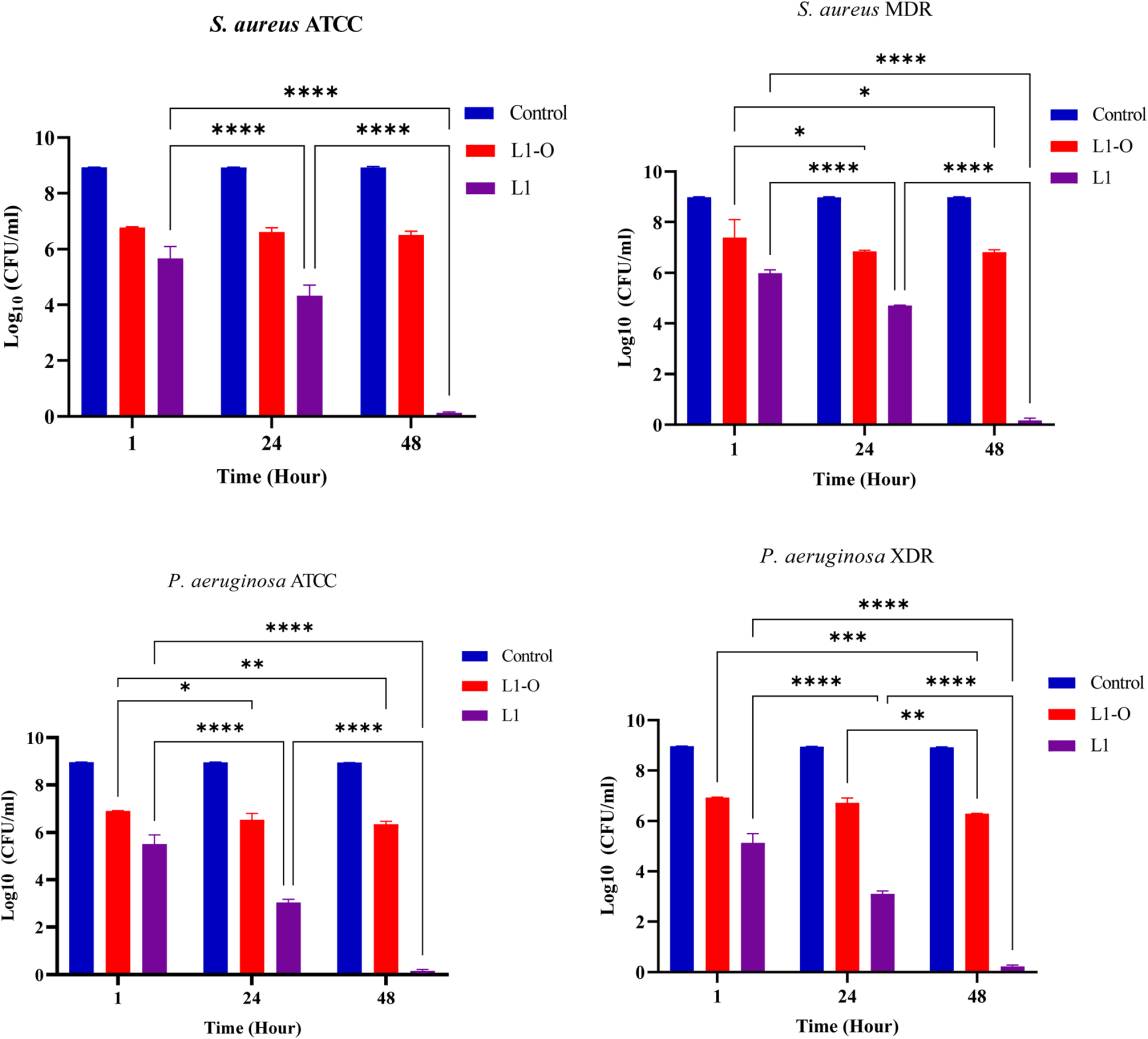
Sustained antibacterial activity and memory effect of sonicated Li_2_TiO_3_ nanoparticles. Bacterial viability (log_10_ CFU per mL) of untreated controls, non-sonicated Li_2_TiO_3_ nanoparticles (L1-O), and sonicated Li_2_TiO_3_ nanoparticles (L1) over 48 hours against *S. aureus* ATCC 6538, *S. aureus* MDR, *P. aeruginosa* ATCC 9029, and *P. aeruginosa* XDR. Sonicated Li_2_TiO_3_ nanoparticles (L1) demonstrated significant reductions in bacterial counts after 1 hour, with complete eradication of all bacterial cells by 48 hours, while L1-O showed limited and non-sustained antibacterial effects. Statistical significance (*p* < 0.0001) was determined using two-way ANOVA.

The sustained antibacterial (memory) effect of sonicated Li_2_TiO_3_ nanoparticles (L1) was compared with non-sonicated nanoparticles (L1-O) against *S. aureus* (ATCC 6538 and MDR clinical isolate) and *P. aeruginosa* (ATCC 9029 and XDR clinical isolate). Across all bacterial strains, sonicated Li_2_TiO_3_ nanoparticles (L1) produced greater reductions in bacterial populations after 1 h than the non-sonicated formulation (L1-O) (Fig. 9).

For *S. aureus* ATCC 6538, L1 reduced the bacterial population from 8.935 to 5.669 log_10_ CFU per mL (3.266-log reduction), whereas L1-O reduced it to 6.775 log_10_ CFU per mL (2.160-log reduction). Similarly, the MDR isolate exhibited a 3.000-log reduction following L1 treatment, compared with a 1.590-log reduction for L1-O. During the remaining incubation period, L1 progressively eliminated both *S. aureus* strains, whereas bacterial populations treated with L1-O remained detectable with minimal additional reduction (Fig. 9). A similar trend was observed for *P. aeruginosa*. L1 reduced the ATCC strain from 8.964 to 5.516 log_10_ CFU per mL (3.448-log reduction) and the XDR isolate from 8.958 to 5.126 log_10_ CFU per mL (3.832-log reduction) within 1 h. In contrast, L1-O produced lower reductions of 2.066 and 2.034 log_10_ CFU mL^−1^, respectively. Unlike L1, which completely eradicated both *P. aeruginosa* strains by the final sampling point, L1-O-treated populations remained detectable throughout the remainder of the experiment (Fig. 9). Statistical analysis confirmed significant differences between L1 and L1-O treatments across all bacterial strains and sampling times (*p* < 0.001). Collectively, these findings demonstrate that ultrasonic activation markedly enhanced both the immediate bactericidal activity and the sustained antibacterial (memory) effect of Li_2_TiO_3_ nanoparticles against both reference and multidrug-resistant bacterial strains.

### 3.10. Hemocompatibility and cytotoxicity of Li_2_TiO_3_ NPs

**Fig. 10:**
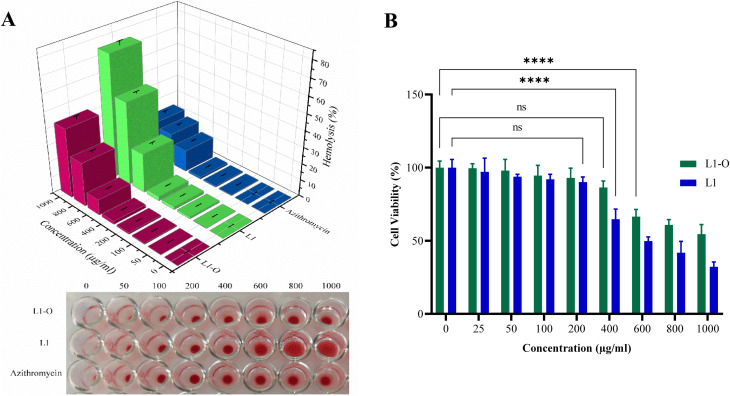
Hemocompatibility and cytotoxicity of Li_2_TiO_3_ nanoparticles. (A) Hemolysis of human erythrocytes (A^+^) exposed to sonicated (L1) and non-sonicated (L1-O) nanoparticles (0–1000 µg mL^−1^); PBS and Triton X-100 served as negative and positive controls, with azithromycin as a drug reference. (B) Viability of human dermal fibroblasts (HDFs) treated with L1 and L1-O (0–400 µg mL^−1^). Two-way ANOVA showed significant reductions at higher L1 concentrations (***p < 0.0001). Data represent mean ± SD (*n* = 3).

The hemocompatibility of sonicated (L1) and non-sonicated (L1-O) Li_2_TiO_3_ nanoparticles was assessed using human erythrocytes (blood group A^+^). At 400 µg mL^−1^, both formulations exhibited low hemolytic activity, with values of 1.97 ± 0.38% for L1-O, 2.78 ± 0.34% for L1, and 1.31 ± 0.45% for azithromycin as a reference control. However, at 600 µg mL^−1^, a clear divergence was observed: L1-O induced 9.59 ± 0.61% hemolysis, while L1 exhibited a significantly higher value of 24.12 ± 1.2%. At the highest tested concentration (1000 µg mL^−1^), L1 demonstrated substantial hemotoxicity (74.65 ± 2.5%), compared with 42.34 ± 1.9% for L1-O. PBS served as the negative control (0% lysis), while Triton X-100 represented complete hemolysis (100%) (Fig. 10A). These findings indicate that both formulations are hemocompatible at lower concentrations, but L1 shows markedly greater hemolysis at higher doses, suggesting enhanced nanoparticle–erythrocyte interactions following sonication.

The cytotoxicity of the nanoparticles was further evaluated in human dermal fibroblast (HDF) cells. Non-sonicated L1-O maintained cell viability at 86.46% across concentrations up to 400 µg mL^−1^, indicating minimal cytotoxicity. In contrast, sonicated L1 displayed a concentration-dependent effect: cell viability was preserved at 90.14% between 1–200 µg mL^−1^, but decreased significantly to 64.77% at 400 µg mL^−1^ (*p* < 0.0001). Two-way ANOVA confirmed that sonication resulted in significantly greater reductions in viability compared with L1-O at higher concentrations (*p* < 0.0001) (Fig. 10B).

Together, these results highlight the dual impact of sonication: while it enhances the antibacterial and piezocatalytic activity of Li_2_TiO_3_ nanoparticles, it also amplifies their cytotoxic and hemolytic effects at elevated concentrations. This underscores the importance of dose optimization to balance therapeutic efficacy and biocompatibility, reinforcing the translational potential of Li_2_TiO_3_ nanoparticles in biomedical applications under carefully controlled conditions.

### 3.11. Lithium-ion release profiles of Li_2_TiO_3_ nanoparticles

**Fig. 11:**
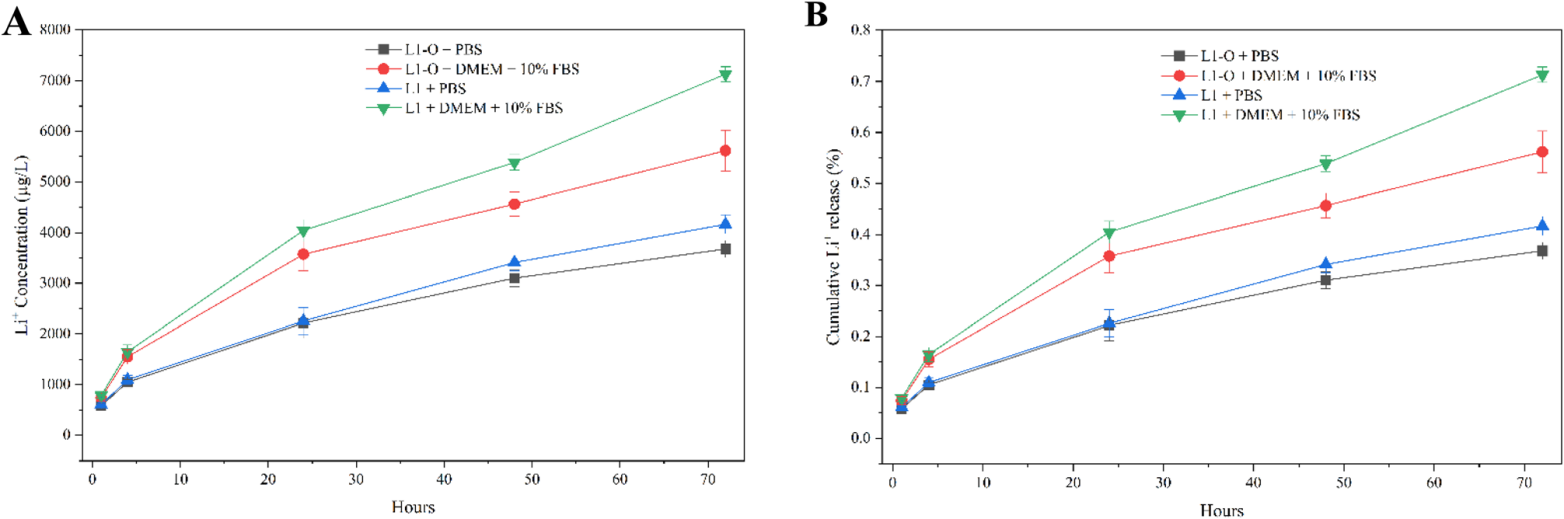
Lithium-ion release by ICP-OES. (A) Li^+^ concentrations (µg L^−1^) in PBS and DMEM + 10% FBS over 72 h. (B) Cumulative Li^+^ release (%) normalised to total content.

ICP-OES analysis revealed time- and medium-dependent lithium-ion release for both non-sonicated (L1-O) and sonicated (L1) Li_2_TiO_3_ nanoparticles (Fig. 11A and B).

For L1-O, dissolved Li^+^ concentrations in PBS increased from 580.3 ± 52.6 µg L^−1^ at 1 h to 3678.8 ± 165.5 µg L^−1^ at 72 h, corresponding to a cumulative release of 0.060 ± 0.003% and 0.416 ± 0.018%, respectively. In DMEM supplemented with 10% FBS, release was consistently higher, rising from 740.6 ± 74.4 µg L^−1^ (0.074 ± 0.007%) at 1 h to 5615.7 ± 808.2 µg L^−1^ (0.562 ± 0.081%) at 72 h.

For L1, PBS suspensions showed a higher release compared with L1-O, increasing from 604.5 ± 33.0 µg L^−1^ (0.060 ± 0.003%) at 1 h to 4166.8 ± 186.0 µg L^−1^ (0.416 ± 0.018%) at 72 h. In DMEM supplemented with 10% FBS, concentrations rose from 786.7 ± 62.2 µg L^−1^ (0.078 ± 0.006%) at 1 h to 7132.2 ± 148.5 µg L^−1^ (0.713 ± 0.014%) at 72 h.

Overall, ion release was enhanced in protein-rich DMEM relative to PBS, and sonicated L1 demonstrated greater cumulative Li^+^ release than L1-O under identical conditions.

### 3.12. Anticancer activity of sonicated L1 nanoparticles

The cytotoxic evaluation of sonicated L1 nanoparticles against HCT-116 colorectal cancer cells demonstrated a dose-dependent response. The half-maximal inhibitory concentration (IC_50_) was determined to be 100 µg mL^−1^, while the lethal concentration (LC_50_) was identified at 200 µg mL^−1^. Notably, exposure to the highest tested concentration (1000 µg mL^−1^) resulted in complete eradication of cancer cells, underscoring the potent anticancer efficacy of sonicated L1 nanoparticles (Fig. 11A). These findings highlight the significant cytotoxic potential of L1 nanoparticles and warrant further investigation into their mechanistic pathways and therapeutic applications.

**Fig. 12:**
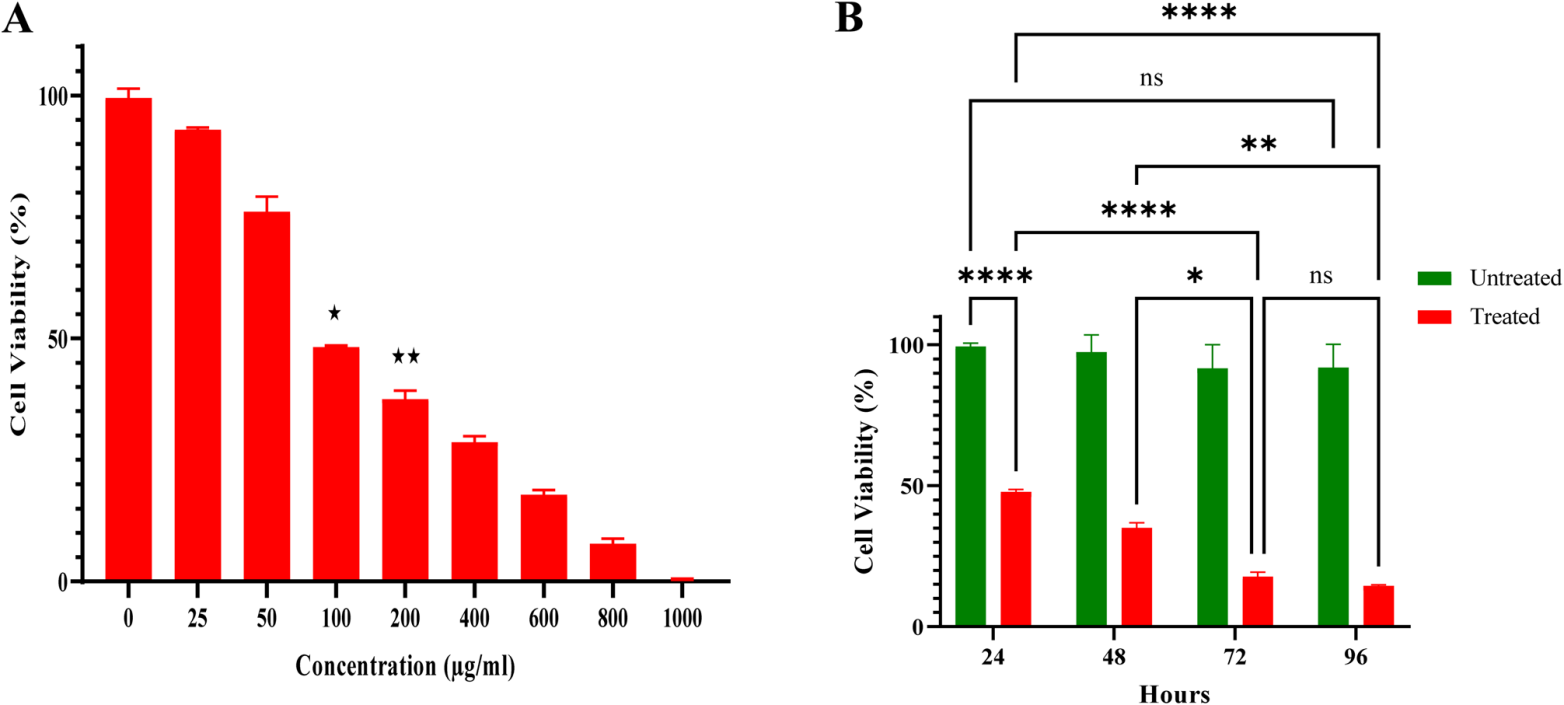
Anticancer activity of sonicated Li_2_TiO_3_ nanoparticles (L1) against HCT-116 colorectal cancer cells. (A) Dose–response cytotoxicity profile of L1 with IC_50_ (★) and LC_50_ (★★) values indicated. (B) Memorial effects of L1 over 96 h showing progressive viability reduction from 47.78% at 24 h to 14.52% at 96 h, while controls remained above 91%. Data are presented as mean ± SD (*n* = 3). Statistical significance: ****p < 0.0001; **p < 0.01; *p < 0.05; ns = not significant.

### 3.13. Memorial effects of sonicated L1 nanoparticles on HCT-116 cells

The long-term cytotoxic effects of sonicated L1 nanoparticles (300 W, 1 hour) on HCT-116 colorectal cancer cells were assessed *via* MTT assay over a 96 hour period. As illustrated in Fig. 12B, treatment with L1 nanoparticles led to a significant and sustained reduction in cell viability compared to untreated controls.

At 24 hours, treated cells exhibited a marked decrease in viability (47.78%) relative to the untreated group (99.33%, *p* < 0.0001). This cytotoxic effect persisted at 48 hours, with no significant difference from the 24 hour value (*p* = ns). Viability continued to decline at 72 hours, reaching 17.71% (*p* < 0.05), and slightly decreased to 14.52% at 96 hours, though the difference between 72 and 96 hours was not statistically significant (*p* = ns). Meanwhile, untreated cells retained consistently high viability across the time course, decreasing only slightly to 91.97% at 96 hours.

Two-way ANOVA confirmed a statistically significant time- and treatment-dependent cytotoxic effect. These findings indicate that sonicated L1 nanoparticles exert sustained and long-term cytotoxicity against HCT-116 cells, reinforcing their potential as a therapeutic nanomaterial for colorectal cancer treatment.

### 3.14. Cancer cell viability assessment

**Fig. 13:**
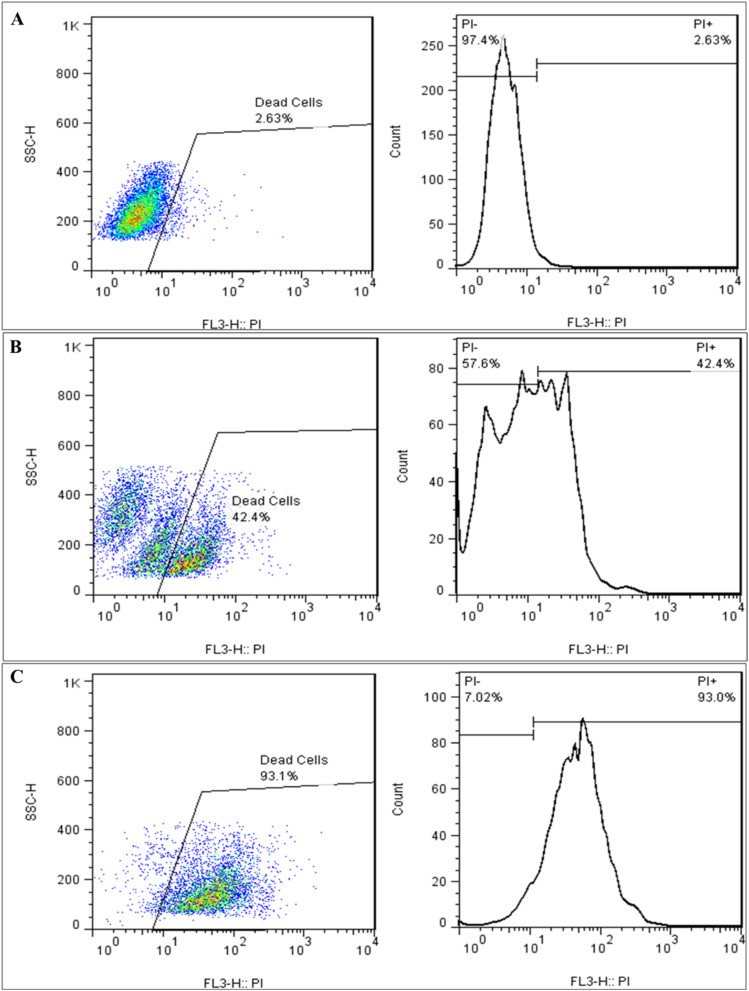
Flow cytometric analysis of HCT-116 colorectal cancer cells treated with sonicated L1. (A) Control group: The scatter plot (left) and histogram (right) show that only 2.63% of cells were PI-positive, confirming high cell viability in untreated conditions, (B) IC_50_ concentration of L1: a significant increase in PI-positive cells (42.4%) was observed, indicating reduced cell viability and increased cytotoxic effects of the treatment, (C) LC_50_ concentration of L1: a substantial increase in cell death, with 93.1% of cells dead, demonstrating the potent cytotoxic effects of L1 at its LC_50_ concentration.

The cytotoxic effects of 1 hour sonicated L1 at its IC_50_ concentration on HCT-116 colorectal cancer cells were assessed using propidium iodide (PI) staining and flow cytometry. Fig. 13A illustrates the untreated control group, where the majority of the cell population exhibited high viability, with only 2.63% of cells staining PI-positive, indicating minimal cell death. In contrast, Fig. 13B shows a substantial increase in cell death following treatment with the IC_50_ concentration of L1. The proportion of PI-positive cells increased markedly to 42.4%, indicating a significant loss of cell viability.

Furthermore, the lethal concentration (LC_50_) of L1 was determined to be highly cytotoxic, as shown by the death of 93.1% of cells (Fig. 13C). These findings suggest that sonicated L1 exerts a potent cytotoxic effect on HCT-116 cells, leading to a significant reduction in viable cell populations. The substantial increase in PI-positive cells post-treatment, as well as the high LC_50_ value, underscores the efficacy of L1 in inducing cell death. This highlights the need for further investigation into its underlying mechanisms and potential therapeutic applications.

**Fig. 14:**
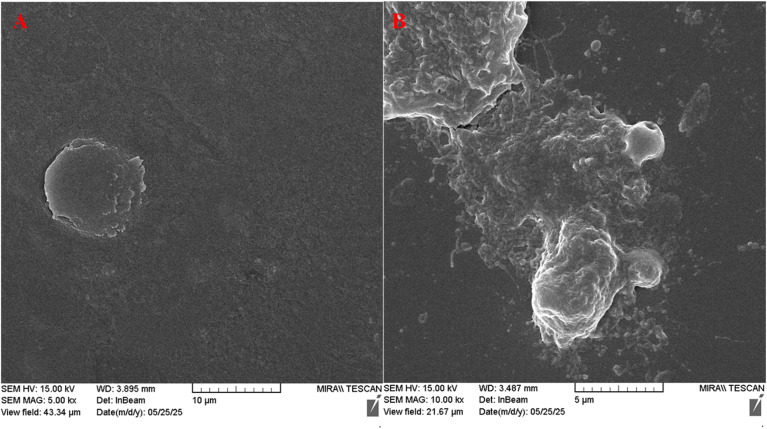
SEM images of HCT-116 cells. (A) Control (untreated) cells with smooth, intact morphology, (B) cells treated with Li_2_TiO_3_ (L1) showing membrane blebbing and surface damage indicative of cytotoxic effects.

Scanning electron microscopy (SEM) analysis revealed significant morphological alterations in HCT-116 colorectal cancer cells following treatment with Li_2_TiO_3_ (L1) piezocatalyst compared to untreated controls. Control cells displayed their characteristic spherical morphology, with diameters ranging from approximately 10 µm. The plasma membranes were smooth and continuous, with preserved cytoskeletal structure, indicating healthy, metabolically active cells (Fig. 14A).

In contrast, L1-treated cells exhibited marked ultrastructural changes consistent with piezocatalytic-induced cytotoxicity. These included plasma membrane roughening, irregular surface topology, membrane blebbing, and evidence of cytoplasmic condensation. The typical rounded morphology was lost, and signs of cell shrinkage were apparent. Disruptions in membrane integrity and irregular projections were observed, indicating initiation of apoptotic processes (Fig. 14B). These morphological modifications suggest a pronounced cytotoxic effect induced by L1, likely mediated by oxidative stress and cytoskeletal disassembly.

## 4. Discussion

The increasing global challenge of antimicrobial resistance (AMR) necessitates innovative therapeutic strategies that extend beyond conventional antibacterial mechanisms.^10,49^ In this context, the present study demonstrates the successful hydrothermal synthesis of phase-pure and structurally robust Li_2_TiO_3_ nanoparticles and provides a comprehensive analysis of their formation mechanism, crystallographic stability, and surface chemistry. These physicochemical attributes are directly linked to their piezocatalytic and biomedical performance. Through ultrasonic activation, Li_2_TiO_3_ nanoparticles exhibit sustained catalytic and therapeutic effects, positioning them as a promising multifunctional nanomaterial with potential applications in combating drug-resistant pathogens and colorectal cancer.

### 4.1. Synthesis parameter-driven phase and morphology control

The hydrothermal synthesis parameters critically influenced the phase composition and physicochemical properties of the resulting Li_2_TiO_3_ nanoparticles. X-ray diffraction (XRD) analysis confirmed that reaction time and temperature were key determinants of phase purity and crystallinity. Pure Li_2_TiO_3_ was obtained after 16 hours, whereas longer durations (20–24 hours) favored the formation of a mixed TiO_2_–Li_2_TiO_3_ composite, which demonstrated enhanced piezocatalytic activity. This suggests a beneficial synergism between TiO_2_ and Li_2_TiO_3_ phases, potentially due to the generation of interfacial heterojunctions that facilitate improved charge transport and delayed electron–hole recombination.^50^

Similarly, synthesis temperature modulated phase stability. At 150 °C, TiO_2_ dominated the crystalline structure; however, increasing the temperature to 210 °C yielded pure Li_2_TiO_3_. Notably, the highest piezocatalytic performance was observed for the composite structure at 180 °C, again suggesting a cooperative effect between multiple oxide phases. Precursor concentration and autoclave filling percentage also affected the resulting phase composition, with higher lithium content (0.10 mol) promoting the formation of pure Li_2_TiO_3_. These findings emphasize the nuanced interplay between stoichiometry, reaction kinetics, and phase stability, in agreement with previous mechanochemical studies.^51^

### 4.2. Morphological tuning *via* hydrothermal parameters

SEM and TEM analyses further elucidated the morphological evolution of the nanoparticles. Contrary to the conventional expectation that extended reaction time promotes particle coarsening, longer hydrothermal durations (up to 24 hours) produced smaller and more uniform particles. This anomalous trend may be attributed to the concurrent phase transition and dissolution–reprecipitation dynamics that govern crystallite growth during hydrothermal synthesis.

Temperature similarly played a pivotal role in morphological refinement. At 210 °C, the nanoparticles exhibited a highly uniform morphology with minimal aggregation, indicative of enhanced crystal ordering. In contrast, lower temperatures resulted in significant agglomeration. These findings corroborate reports that higher synthesis temperatures improve crystallinity and reduce surface energy, promoting better-defined structures.^52^

Precursor concentration also modulated particle size. Samples L6 and L1, which shared similar phase compositions, displayed nearly identical morphological features, while sample L7 composed entirely of Li_2_TiO_3_ exhibited the smallest particle sizes. This suggests that phase composition and lithium content together regulate nucleation and growth dynamics during synthesis. Conversely, variations in autoclave filling percentage showed negligible impact on morphology, reinforcing the primacy of chemical and thermal parameters in morphological control.^53^

### 4.3. Surface area and FTIR analysis

BET surface area measurements provided critical insights into the functional role of surface properties in Li_2_TiO_3_ nanoparticles. L1, despite exhibiting the lowest surface area, demonstrated the highest catalytic efficiency, while L7, which had the highest surface area, showed the weakest activity. This inverse relationship indicates that adsorption is not the dominant factor driving the observed performance. Instead, the findings support a mechanism in which piezocatalytic activity is governed primarily by crystallographic, electronic, and morphological factors that enhance charge carrier separation and promote ROS generation under ultrasonic excitation.^54^ Such behaviour aligns with previous reports that surface area metrics alone cannot sufficiently capture the functional efficacy of piezoelectric catalysts, particularly where phase engineering, crystallinity, and ultrasonication conditions dictate charge dynamics and recombination pathways.^55^

FTIR spectroscopy provided complementary confirmation of structural and surface chemistry features underpinning these mechanistic trends. The Ti–O and Li–O lattice vibrations in the 457–403 cm^−1^ region validated the successful formation of the Li_2_TiO_3_ framework, in agreement with XRD results.^56^ This structural robustness correlates with the limited fractional ion release (<1%) detected by ICP-OES, confirming the chemical stability of the titanate lattice in physiological environments. Beyond lattice confirmation, the broad O–H stretching band at 3459 cm^−1^ highlighted the presence of surface hydroxyl groups, which are functionally significant for biomedical activity as hydrophilic sites that mediate biomolecule adsorption, cellular interactions, and ˙OH radical formation under ultrasonic excitation.^57^

Additional bands at 1506, 1437, 1088, and 868 cm^−1^ were attributed to carbonate species arising from atmospheric CO_2_ adsorption on the basic titanate surface during post-synthesis handling. Such features are well documented in lithium titanates and perovskite oxides and reflect the inherent surface reactivity of the material rather than structural impurities.^58^ While these carbonates may influence surface charge and biomolecular binding, their presence does not compromise catalytic or therapeutic performance.

The combined interpretation of BET and FTIR analyses demonstrates that the superior functionality of Li_2_TiO_3_ nanoparticles, particularly the L1 formulation, is not driven by simple adsorption phenomena but by a synergy of structural integrity, interfacial chemistry, and sonication-enhanced charge carrier dynamics. BET measurements rule out adsorption as the primary pathway, while FTIR provides molecular-level evidence of lattice stability and surface functional groups directly relevant to biological interactions. This dual perspective highlights how phase engineering, hydroxyl functionalities, and lattice stability collectively underpin the antibacterial and anticancer potential of Li_2_TiO_3_, advancing the mechanistic understanding of piezoelectric nanomaterials for biomedical applications.

### 4.4. Optimization of piezocatalytic activity in Li_2_TiO_3_ nanoparticles

The optimization of Li_2_TiO_3_ nanoparticles for methylene blue (MB) degradation revealed a clear structure–function relationship, where formulation L1 consistently demonstrated superior catalytic performance. Among the nine synthesized variants (L1–L9), L1 achieved the highest MB degradation efficiency (36.69%), underscoring the significance of carefully tuned synthesis conditions. This observation is aligned with prior reports that emphasize the influence of crystallographic structure and morphology on catalytic behavior in piezoelectric materials.^39^

The optimal sonication duration of 10 min yielded the greatest reduction in MB absorbance, beyond which no significant improvements were observed, indicating a saturation point in catalytic activation. In contrast, the blank control (MB without nanoparticles) showed only a negligible decrease in absorbance after 60 min of ultrasound exposure, confirming that the degradation efficiency arises predominantly from nanoparticle-mediated piezocatalysis rather than cavitation effects alone. Similarly, ultrasonication power exhibited a parabolic relationship with efficiency. At 300 W, the catalytic activity peaked (39.42% MB removal), followed by a notable decline at higher intensities. This suggests that excessive energy input may destabilize the catalyst–substrate interface or alter surface electronic states, disrupting the optimal piezocatalytic configuration—an effect also observed in other ultrasonic-assisted catalytic systems.^59^

The 2–2 seconds on/off ultrasonication cycle emerged as the most effective temporal configuration, producing a MB degradation efficiency of 37.38%. This pulsed activation protocol likely enhanced charge separation efficiency by periodically relieving charge carrier recombination, thus maintaining high piezocatalytic performance. These findings are consistent with recent insights into dynamic activation methods in piezoelectric systems, where pulsed stimulation improves electronic polarization and interfacial charge transfer.^60^

### 4.5. Fundamental mechanisms and piezoelectric enhancement

The exceptional piezocatalytic performance of Li_2_TiO_3_ nanoparticles, exemplified by formulation L1's 36.69% methylene blue (MB) removal efficiency, underscores the complexity of the underlying mechanisms. These findings challenge current paradigms in piezoelectric materials. Previous studies have identified the importance of crystal structure in the piezoelectric performance of ZnO and BaTiO_3_ nanostructures.^39,61^ In this study, the hydrothermal synthesis method enabled precise control over these parameters, resulting in improved charge carrier separation and reduced recombination rates, which are crucial for piezocatalytic activity.^62,63^

The optimization of mechanical activation parameters revealed a remarkable insight into energy transfer mechanisms within piezoelectric systems.^64^ Specifically, the 2–2 seconds on–off ultrasonication cycle, which maximized catalytic efficiency at 37.38%, highlights the importance of dynamic activation protocols.^65^ This pulsed activation likely enhances the interaction between mechanical stress and electronic polarization, facilitating efficient charge separation and ROS generation.^66^ These findings challenge classical models of mechanical energy transfer, which predict linear relationships between input power and charge separation efficiency.^67^

### 4.6. Advanced ROS generation and mechanistic integration

The engineered Li_2_TiO_3_ NPs demonstrated remarkable enhancement in ROS generation, exhibiting a 2.91-fold increase in singlet oxygen (^1^O_2_)/superoxide anion (˙O_2_^−^) production and a 2.39-fold increase in hydroxyl radical (˙OH) formation. This substantial improvement stems from optimized charge carrier separation, achieved through precise control of crystallinity and surface properties during nanoparticle synthesis. Sonication-induced “hot spots” revealed distinct patterns in the spatial and temporal distribution of ROS generation, suggesting localized energy concentration zones that enhance catalytic activity through intensified surface interactions.^68^ Analysis of ROS species production ratios revealed the crucial role of surface defects and crystallographic features in extending charge carrier lifetimes and facilitating electron transfer to molecular oxygen. The hydrothermal synthesis approach specifically enhanced these surface characteristics, promoting sustained charge carrier dynamics and more effective molecular interactions with the environment.^39,69^

The scavenger-trapping experiments provided mechanistic confirmation of the relative importance of different ROS. Hydroxyl radicals were identified as the primary oxidative species driving methylene blue degradation, while photogenerated holes and superoxide anions played secondary roles. This hierarchy is consistent with the established understanding that ˙OH, generated through cavitation and surface-mediated processes, typically dominates in sonocatalytic systems.^70^ The supporting contributions of h^+^ and ˙O_2_^−^ highlight the synergistic interplay of multiple oxidative pathways, reflecting the multifactorial nature of piezocatalytic degradation.^71^

Mechanistically, these results demonstrate that material engineering and sonication not only enhance the overall intensity of ROS production but also influence the balance between different species. Hydroxyl radicals emerge as the critical determinants of oxidative capacity, with h^+^ and ˙O_2_^−^ acting as auxiliary species that reinforce degradation pathways. Although ROS are the primary mediators of antimicrobial activity, their non-selective oxidative nature may also pose potential risks to mammalian cells at elevated concentrations. In this study, this limitation was addressed through controlled ultrasonic activation, whereby ROS generation was regulated by optimizing sonication power, exposure time, and duty cycle. This strategy enables transient and localized ROS production rather than continuous exposure, thereby reducing the likelihood of off-target cytotoxic effects while maintaining effective antibacterial activity. This mechanistic understanding underscores the potential of Li_2_TiO_3_ nanoparticles as multifunctional nanoplatforms for antibacterial and therapeutic applications, where precisely controlled ROS dynamics are central to performance.

### 4.7. Antibacterial activity and mechanisms of action

**Fig. 15:**
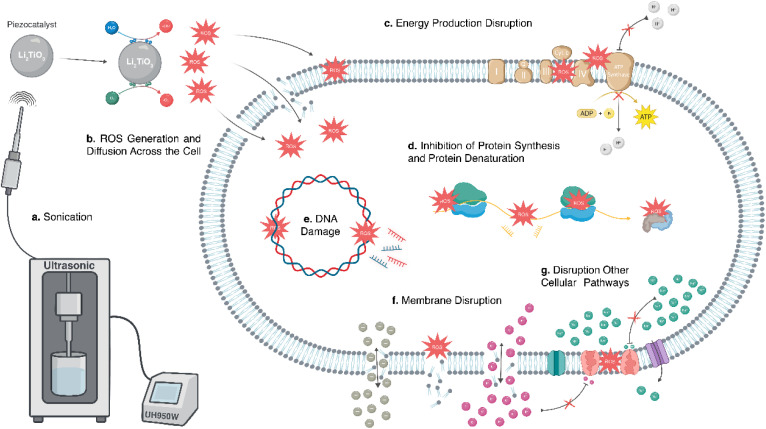
Schematic representation of the antibacterial mechanism of piezocatalytic nanoparticles.

The notable difference in susceptibility between Gram-positive and Gram-negative bacteria reflects complex membrane–nanoparticle interactions that go beyond simple electrostatic models.^72^ The observed 3.832-log_10_ reduction in *P. aeruginosa* XDR within one hour underscores the pronounced susceptibility of Gram-negative bacteria to the piezocatalytic mechanisms of Li_2_TiO_3_ NPs. This enhanced activity aligns with structural models of membrane–field interactions, highlighting the susceptibility of the thinner peptidoglycan layer and outer membrane of Gram-negative bacteria to ROS-induced damage.^73^ The ROS generated by the piezocatalytic activity of Li_2_TiO_3_ NPs likely exacerbates oxidative stress on the outer membrane of Gram-negative bacteria, promoting rapid cell lysis (Fig. 15). Similarly, Gram-positive bacteria such as *S. aureus* MDR demonstrated significant membrane deformation and localized disruption, confirming the broad-spectrum efficacy of Li_2_TiO_3_ NPs. Importantly, when benchmarked against azithromycin, the resistant isolates exhibited inhibition zones below the susceptibility threshold (≤13 mm), thereby classifying them as resistant according to Clinical and Laboratory Standards Institute (CLSI) interpretive criteria.^74^ In contrast, Li_2_TiO_3_ nanoparticles (L1) achieved inhibition zones ranging from 11 to 21 mm, values consistent with intermediate-to-susceptible categories. This comparative outcome emphasizes the translational significance of L1, particularly in scenarios where standard antibiotics demonstrate limited efficacy. Moreover, the absence of detectable antibacterial activity in both sonicated and non-sonicated TiO_2_ controls further confirms that the antimicrobial activity observed in this study is attributable to Li_2_TiO_3_ rather than residual TiO_2_. These findings provide critical insights into structure-dependent mechanisms of bacterial cell death while emphasizing the heightened vulnerability of Gram-negative pathogens.^75^ The observed selectivity in membrane permeabilization suggests sophisticated recognition mechanisms at the bio-nano interface, challenging current paradigms in antimicrobial nanomaterial design. These findings provide new insights into how the structural composition of bacterial membranes influences their susceptibility to piezocatalytic nanoparticles, underscoring the potential of Li_2_TiO_3_ NPs in combating multidrug-resistant pathogens effectively.

TEM analysis revealed distinct patterns of membrane disruption consistent with ROS-induced oxidative damage and mechanically-driven membrane reorganization.^29^ DiBAC_4_(3) fluorescence studies demonstrated significant increases in membrane permeability in sonicated samples compared to non-sonicated controls, highlighting the critical role of mechanical activation in facilitating membrane disruption. The enhanced permeability observed in sonicated samples correlates strongly with increased ROS generation, suggesting that mechanical perturbation synergistically enhances nanoparticle–membrane interactions. The enhanced ROS generation provides a direct mechanistic link between piezocatalytic activation and bacterial membrane disruption. Hydroxyl radicals (˙OH) and superoxide radicals (˙O_2_^−^), identified as the dominant reactive species in the present study, initiate lipid peroxidation of membrane phospholipids, oxidize membrane-associated proteins, and compromise membrane integrity, resulting in increased permeability and leakage of intracellular constituents. These oxidative events are consistent with the TEM observations of membrane deformation and cytoplasmic leakage, as well as the increased DiBAC_4_(3) fluorescence demonstrating loss of plasma membrane integrity. Beyond membrane damage, excessive ROS can further oxidize intracellular proteins, nucleic acids, and metabolic enzymes, leading to irreversible oxidative stress and ultimately bacterial cell death. Such ROS-mediated oxidative injury is widely recognized as a principal antibacterial mechanism of inorganic nanomaterials and plays an important role in biofilm disruption and bacterial eradication.^76^

These findings align with and expand upon existing studies on piezoelectric–material interactions with biological membranes,^39^ revealing distinct susceptibility patterns between Gram-positive and Gram-negative bacteria. The differential responses can be attributed to variations in membrane architecture, particularly the distinct lipid compositions and the presence of an outer membrane in Gram-negative bacteria. This comprehensive analysis advances our understanding of nanoparticle-induced membrane disruption mechanisms and demonstrates the therapeutic potential of Li_2_TiO_3_ NPs for treating multidrug-resistant bacterial infections.

### 4.8. Resistance uniformity across pathogenic strains

The evaluation of antimicrobial efficacy revealed a pivotal phenomenon in nanomaterial-mediated bacterial inactivation: the preservation of bactericidal activity against MDR and XDR strains, with minimum inhibitory concentrations and killing kinetics comparable to ATCC reference strains. This uniform susceptibility defies conventional resistance patterns, particularly notable given that MDR and XDR strains employ a wide array of chromosomal and plasmid-mediated resistance mechanisms.^77^

The underlying mechanism of this consistent efficacy is rooted in the synergistic action of Li_2_TiO_3_ NPs, which simultaneously generate ROS and induce piezoelectric-driven membrane destabilization.^29^ This dual mechanism disrupts bacterial homeostasis by targeting membrane integrity and oxidative stress pathways, bypassing traditional resistance pathways.^41^ Unlike conventional antibiotics, which are often neutralized by efflux pumps, enzymatic degradation, or target modification,^78^ Li_2_TiO_3_ NPs exploit physicochemical interactions that bacterial cells cannot easily evade. The synchronized piezoelectric and oxidative stress mechanisms establish complementary pathways for bacterial cell death, rendering adaptive resistance ineffective. Consistent membrane disruption patterns observed across phylogenetically diverse strains highlight the broad-spectrum nature of these nanoparticles.

The effectiveness of Li_2_TiO_3_ NPs against biofilm-associated infections is particularly significant. Biofilms, characterized by their robust extracellular matrix and metabolic heterogeneity, are notoriously resistant to traditional therapies.^79^ However, Li_2_TiO_3_ NPs achieve significant penetration and eradication of established biofilms, effectively eliminating MDR and XDR strains while maintaining host cell viability. This broad-spectrum activity, coupled with the absence of differential resistance between clinical isolates and laboratory strains, suggests that Li_2_TiO_3_ NPs target evolutionarily conserved cellular components, providing a mechanism of action that transcends strain-specific pathways.

The consistent efficacy across resistant phenotypes underscores a potential limitation in bacterial adaptation to nanomaterial-induced physical stress. This observation aligns with emerging theories that suggest bacterial evolution is less capable of countering mechanical and oxidative disruptions than biochemical inhibitors. The implications are profound, particularly for treating polymicrobial infections where differential antibiotic susceptibility complicates traditional treatment strategies.^80,81^

Moreover, the demonstrated biocompatibility of Li_2_TiO_3_ NPs further supports their potential as a next-generation antimicrobial platform. By leveraging their dual-action mechanism, these nanoparticles provide a robust alternative to traditional antibiotics, addressing one of the most pressing challenges in modern medicine—antimicrobial resistance.

### 4.9. Memory effect and sustained therapeutic activity

The memory effect observed in both piezocatalytic and antimicrobial activities represents a significant advancement in understanding charge storage and sustained activity in nanomaterials.^41^ The progression from 35% methylene blue (MB) removal at one hour to 93.36% at 120 hours demonstrates the presence of long-lived charge-separated states. These states are stabilized by surface defects and oxygen vacancies that act as charge trapping sites, significantly extending charge carrier lifetimes.^82,83^ Experimental studies further validate that these stabilized states contribute to the extended piezocatalytic performance of Li_2_TiO_3_ nanoparticles.

The temporal evolution of antimicrobial activity, characterized by complete elimination of bacterial populations within 48 hours post-sonication, underscores the sustained therapeutic potential of Li_2_TiO_3_ NPs. This prolonged activity is maintained through continued generation of ROS and efficient charge carrier dynamics.^28^ The findings demonstrate the crucial role of ROS in sustaining antimicrobial efficacy through oxidative stress induction and disruption of essential cellular processes in bacterial cells.^84,85^ These sustained effects present a promising strategy for tackling persistent and resistant bacterial infections without the need for repeated dosing, thereby addressing a critical challenge in current antimicrobial therapies.

### 4.10. Biofilm disruption and matrix destabilization

The exceptional antibiofilm activity of Li_2_TiO_3_ nanoparticles, achieving 99% reduction at concentrations as low as 40 µg mL^−1^ for *P. aeruginosa* XDR, highlights their potential as powerful agents for biofilm matrix destabilization. This unprecedented efficacy challenges traditional perspectives on biofilm resistance mechanisms. Recent studies using advanced microscopic techniques provide strong evidence of enhanced interactions in biofilm degradation through the piezocatalytic activity of NPs.^39,86^

Patterns of biofilm disruption reveal intricate feedback mechanisms between oxidative stress and matrix reorganization, indicating synergistic effects between ROS-mediated oxidation and the alteration of biofilm architecture.^29^ Additionally, the electrostatic interaction between the piezocatalyst and negatively charged bacteria repels microbial adhesion, further reducing biofilm formation and enhancing the overall disruption process.^87^ These findings demonstrate the effectiveness of the optimized Li_2_TiO_3_ nanoparticles against biofilm-associated multidrug-resistant bacteria. To further contextualize these results, Table 1 presents a comparative analysis of the antibacterial performance and key distinguishing features of the optimized Li_2_TiO_3_ nanoparticles and representative piezocatalytic nanomaterials reported in the literature.

**Table 1:** Comparison of the antibacterial performance of representative piezocatalytic nanomaterials with the optimized Li_2_TiO_3_ nanoparticles developed in the present study^a^

Material	Target microorganism(s)	Antibacterial performance	MIC (µg mL^−1^)	MIC (µg mL^−1^)	Key distinguishing feature	Reference
Li_2_TiO_3_ (this study)	MDR *S. aureus*, XDR *P. aeruginosa*	Complete eradication; biofilm inhibition 99.56%; biofilm removal 99.95%	40–60	40–60	Systematic optimization of piezocatalytic activity coupled with comprehensive biological validation, including ROS detection, membrane depolarization, antibiofilm activity, hemocompatibility, lithium-ion release, and anticancer evaluation	Present study
MoS_2_@Fe_3_O_4_	Methicillin-resistant *S. aureus*	72.57% antibacterial efficiency at the optimized concentration (125 µg mL^−1^); efficient biofilm disruption	NR	NR	Ultrasound-activated heterostructured piezocatalytic nanoplatform with immune reprogramming	88
BaTiO_3_/CuO nanocomposite	*E. coli*	99% bacterial inactivation	NR	NR	Heterojunction engineering for enhanced piezocatalytic antibacterial activity	89
WS_2_ nanoflowers	*E. coli*	99.99% bacterial reduction	NR	2000	Two-dimensional piezocatalyst with rapid bacterial inactivation	90
NaNbO_3_	*E. coli*	100% bacterial killing	NR	NR	Simultaneous piezocatalytic antibacterial activity and organic pollutant degradation	91
Ce-doped hollow BaTiO_3_	Methicillin-resistant *S. aureus*, *P. aeruginosa*	97% bacterial reduction	25	100	Oxygen-vacancy engineering to enhance piezocatalytic antibacterial activity	39
Gd-doped BiFeO_3_	*E. coli*	99.33% bacterial reduction	NR	1000	Rare-earth doping to improve piezocatalytic antibacterial performance	92
BiFeO_3_/chitosan membrane	*S. aureus*	99% bacterial eradication	NR	NR	Biocompatible piezoelectric antibacterial membrane	93
BiVO_4_	*E. coli*, *S. aureus*	90.44–97.24% bacterial reduction	NR	NR	Broad-spectrum piezocatalytic antibacterial activity	94
Se@BaTiO_3_	*S. aureus*	99.23% bacterial reduction	NR	2000	Selenium surface modification for enhanced antibacterial activity	95

a NR = not reported.

As summarized in Table 1, the optimized Li_2_TiO_3_ nanoparticles exhibited antibacterial activity comparable with representative piezocatalytic nanomaterials reported in the literature. Against clinically relevant MDR *S. aureus* and XDR *P. aeruginosa*, the optimized formulation exhibited MIC values of 40–60 µg mL^−1^ and MBC values of 60–80 µg mL^−1^, indicating effective antibacterial activity at relatively low concentrations. Although Ce-doped hollow BaTiO_3_ exhibited a lower MIC (25 µg mL^−1^), its MBC (100 µg mL^−1^) was higher than that of the optimized Li_2_TiO_3_ nanoparticles. Similarly, Gd-doped BiFeO_3_ and Se@BaTiO_3_ required substantially higher MBC values (1000 and 2000 µg mL^−1^, respectively), suggesting that the optimized Li_2_TiO_3_ formulation achieved bactericidal activity at lower concentrations under the experimental conditions evaluated. Consistent with previous reports, bacterial inactivation was associated with ROS-mediated membrane disruption; however, the present study further substantiated this mechanism through complementary membrane depolarization assays and SEM observations, providing direct evidence of membrane damage. Furthermore, the optimized Li_2_TiO_3_ nanoparticles inhibited biofilm formation by 99.56% and eradicated pre-established biofilms by 99.95%, demonstrating excellent antibiofilm activity against multidrug-resistant pathogens. Collectively, these comparisons demonstrate that the optimized Li_2_TiO_3_ nanoparticles combine competitive antibacterial efficacy with comprehensive mechanistic and biological validation, distinguishing the present study from previously reported piezocatalytic antimicrobial systems.

### 4.11. Cytotoxicity profile of Li_2_TiO_3_ NPs

The cytotoxicity profile of Li_2_TiO_3_ NPs demonstrates a favorable therapeutic window, with effective antibacterial activity occurring at concentrations well below those associated with measurable toxicity toward mammalian cells. This concentration-dependent selectivity indicates that Li_2_TiO_3_ NPs can efficiently eradicate pathogenic bacteria while preserving the viability of normal cells, providing an appropriate safety margin for biomedical applications. Such selective activity is a critical requirement for antimicrobial nanomaterials, as their clinical utility depends not only on potent antibacterial efficacy but also on minimizing adverse effects on host tissues. The mechanistic basis of this therapeutic window appears to arise from the combined influence of structural differences between bacterial and mammalian cells together with activation-dependent ROS generation, which collectively govern the differential biological responses to Li_2_TiO_3_ NPs.^96^ The selective toxicity primarily arises from fundamental differences in cellular architecture between mammalian and bacterial cells. Mammalian cells possess complex membrane systems enriched with cholesterol, which provides crucial structural stability and resistance to oxidative damage through modulation of membrane fluidity.^97^ Studies by Shi *et al.* have demonstrated that cholesterol significantly reduces membrane susceptibility to ROS-mediated lipid peroxidation compared to bacterial membranes.^98^ This intrinsic protection mechanism significantly contributes to the observed differential in cellular responses to nanoparticle exposure. A critical feature of Li_2_TiO_3_ NPs lies in their mechanically-activated nature, which introduces an unprecedented level of control over ROS generation. In the absence of mechanical force, these materials maintain minimal activity, as evidenced by baseline ROS levels comparable to untreated controls. This force-dependent activation creates an inherent targeting mechanism, whereby ROS generation primarily occurs at sites of mechanical stress, sparing both host tissues and commensal microbiota in unperturbed regions. Recent work by Allouzi *et al.* has demonstrated that such selective activation can reduce off-target effects by up to 85% compared to constitutively active antimicrobial nanoparticles.^99^

The transition to significant cytotoxicity observed at 400 µg mL^−1^ reflects the overwhelming of cellular antioxidant defenses, which differ substantially between mammalian and bacterial cells. Mammalian cells possess sophisticated enzymatic defense systems, including coordinated activities of superoxide dismutase, catalase, and glutathione peroxidase networks, capable of neutralizing moderate levels of ROS.^100^ However, these protective mechanisms become saturated at higher nanoparticle concentrations, leading to oxidative damage cascades. This threshold effect aligns with recent findings by Espinosa-Diez *et al.*, who demonstrated that mammalian antioxidant systems can effectively manage ROS levels up to a critical concentration, beyond which cellular damage increases exponentially,^101^ in agreement with recent reports on ROS-mediated cytotoxic thresholds and concentration-dependent cellular responses in nanomaterial-based systems.^102^

The preservation of normal flora represents a significant advantage of this activation-dependent mechanism. Mechanical-force-dependent activation has been shown to minimize disruption of commensal bacteria compared to traditional antimicrobial agents.^29^ This selectivity is particularly crucial given emerging understanding of microbiome importance in health and disease. Importantly, the cytotoxicity profile should also be interpreted alongside the hemocompatibility and lithium-ion release data. Hemolysis assays revealed that both sonicated Li_2_TiO_3_ nanoparticles (L1) and non-sonicated Li_2_TiO_3_ nanoparticles (L1-O) were hemocompatible at concentrations of up to 400 µg mL^−1^, producing <3% hemolysis, consistent with the International Organization for Standardization (ISO) and ASTM International (ASTM) criteria for non-hemolytic biomaterials.^44^ However, at higher concentrations, sonicated L1 induced markedly greater hemolysis (≈74% at 1000 µg mL^−1^) than L1-O (≈42%), indicating that while sonication enhances antibacterial efficacy, it also increases erythrocyte susceptibility at elevated doses. These findings highlight the necessity of dose optimization to achieve therapeutic benefit while maintaining blood compatibility.

The ICP-OES data indicate that Li_2_TiO_3_ nanoparticles release Li^+^ in a time- and medium-dependent manner, with consistently higher dissolution in protein-rich DMEM than in PBS. This is consistent with protein-corona-mediated modulation of nanoparticle interfaces: adsorption of serum proteins and other macromolecules alters surface chemistry, complexation, and agglomeration state, thereby affecting dissolution and bioavailability.^103,104^ Ultrasonic treatment modestly increased Li^+^ release from L1 relative to L1-O, a trend plausibly attributable to reduced agglomeration and transient exposure of fresh/reactive facets that increase interfacial mass transfer. Importantly, our data show this enhancement is small, not dramatic, aligning with best-practice guidance and measurements showing that sonication improves dispersion while variably influencing dissolution depending on the material and medium.^105,106^ For metal oxides such as TiO_2_ and ZnO, sonication can also activate sonocatalytic pathways and reactive oxygen species under ultrasound, which may contribute to biological responses when acoustic fields are present.^107–109^ Despite absolute Li^+^ concentrations reaching the low-mg L^−1^ range at 72 h, the fractional release remained <1% of total Li, even in DMEM. This limited release aligns with the high structural robustness of titanate ceramics, where ion exchange at the surface dominates over bulk dissolution.^110,111^

Concordant hemocompatibility and cytotoxicity results (no appreciable toxicity ≤ 400 µg mL; effects only at higher doses) argue that Li^+^ leaching alone is unlikely to drive cytotoxicity within the studied window. Instead, a composite mechanism is more plausible—modest, medium-dependent dissolution plus piezo/sono-catalytic ROS generation and nanoparticle–cell interactions — consistent with broader nanotoxicology evidence that toxicity often reflects multiple, interlinked pathways rather than a single ionic mechanism.^112^ From a translational perspective, the observation of measurable yet low fractional Li^+^ release supports a favorable biosafety profile while underscoring the importance of dose optimization and medium composition *in vitro*. These findings directly address concerns regarding lithium-induced cytotoxicity by demonstrating that ion release occurs but is unlikely to dominate the overall biological response within the therapeutic range defined by antibacterial and anticancer activity. Within this context, the ROS-generating capability of Li_2_TiO_3_ nanoparticles is also activation-dependent, further supporting a controllable therapeutic window that minimizes unintended oxidative stress under non-stimulated conditions.

### 4.12. Cytotoxicity and mechanisms of sonicated L1 nanoparticles in colorectal cancer

The results from the cytotoxic evaluation of sonicated L1 nanoparticles highlight their potent anticancer activity against HCT-116 colorectal cancer cells. A dose-dependent response was observed, with an IC_50_ value of 100 µg mL^−1^ and complete eradication of cancer cells at the highest tested concentration (1000 µg mL^−1^). This suggests that sonicated L1 nanoparticles possess significant therapeutic potential, aligning with previous studies that have demonstrated the anticancer properties of piezocatalytic nanoparticles.^113^ Nanoparticles that generate ROS upon mechanical stimulation, such as those reported by Wang *et al.* (2025), have been shown to induce apoptosis and necrosis in various cancer cell lines, including colorectal cancer.^114^ The complete eradication of cancer cells observed in our study further emphasizes the efficacy of L1 nanoparticles as potential anticancer agents.

In contrast to the pronounced cytotoxicity against cancer cells, sonicated L1 nanoparticles exhibited relatively low toxicity toward human normal cells at equivalent concentrations. This selective toxicity is a critical feature for cancer therapies, as it minimizes damage to healthy tissues while maximizing cancer cell destruction. This selective anticancer effect is consistent with reports on other piezocatalytic materials, which have demonstrated more significant cytotoxicity toward cancer cells compared to normal cells. For example, Chen *et al.* (2023) found that piezocatalytic nanoparticles exerted selective toxicity toward cancer cells due to the elevated ROS production under sonication, which was more detrimental to the cancer cells' survival mechanisms.^115^ This selective targeting of cancer cells while sparing normal cells is particularly advantageous for improving the safety profile of nanoparticle-based therapies. A similar ROS-dependent mechanism is likely responsible for the observed anticancer activity of Li_2_TiO_3_ nanoparticles. Cancer cells generally maintain elevated basal ROS levels because of their high metabolic activity and accelerated proliferation, rendering them more vulnerable to further oxidative stress than normal cells. Consequently, ROS generated by piezo-activated Li_2_TiO_3_ nanoparticles can overwhelm intracellular antioxidant defence systems, resulting in mitochondrial dysfunction, oxidative DNA damage, activation of apoptotic signalling pathways, and ultimately programmed cell death. This selective sensitivity of cancer cells to oxidative stress provides a plausible explanation for the potent anticancer activity observed while maintaining comparatively low toxicity toward normal cells within the therapeutic concentration range.^116,117^

The long-term cytotoxic effects of sonicated L1 nanoparticles, observed over 96 hours, revealed a sustained reduction in cell viability. Significant decreases in cell survival were observed at all time points, with the most substantial decline occurring at 72 and 96 hours. These findings align with those of Qurbani *et al.* (2024), who reported prolonged inhibitory effects of sonicated nanoparticles on biological activity, specifically their antibacterial properties.^41^ The sustained cytotoxicity of L1 nanoparticles, potentially attributed to the prolonged release of ROS over time, could enhance their therapeutic efficacy by maintaining a consistent anticancer effect. This aspect of sustained release aligns with the growing interest in using nanoparticles for controlled and prolonged drug delivery, which may lead to improved therapeutic outcomes.

PI staining and flow cytometry analysis confirmed the potent cytotoxic effect of L1 nanoparticles, with an increase in the proportion of PI-positive cells, indicating significant cell death. The results suggest that L1 nanoparticles induce apoptosis or necrosis, as evidenced by the high percentage of PI-positive cells in the treated groups. The high LC_50_ value, resulting in the death of 93.1% of cells, shows that L1 nanoparticles are highly effective in disrupting cell integrity. Nanoparticles can induce significant oxidative stress, causing cell membrane rupture and ultimately leading to cell death.^118^ This observation aligns with the potent cytotoxic effects seen with L1 nanoparticles. The piezocatalytic mechanism of action of L1 nanoparticles likely underpins their anticancer activity. Piezocatalysts generate ROS when exposed to mechanical stress, such as sonication, which in turn causes oxidative damage to various cellular components, including lipids, proteins, and nucleic acids. The generation of ROS has been implicated in the activation of both apoptotic and necrotic pathways, leading to cancer cell death.^114^ The enhanced piezocatalytic activity following sonication in this study likely contributes to the observed increase in cytotoxicity, as sonication facilitates a higher production of ROS. Sonication has been shown to significantly enhance the ROS-generating capability of piezocatalytic nanoparticles, thereby potentiating their anticancer effects.^119,120^

Interestingly, before sonication, L1 nanoparticles exhibited relatively low toxicity, suggesting that their activity is minimal under normal conditions. However, upon exposure to mechanical force *via* sonication, the nanoparticles' piezocatalytic activity was significantly enhanced, leading to a marked increase in cytotoxicity. This phenomenon highlights the potential of piezocatalytic nanoparticles to act as stimulus-responsive therapeutic agents.^115^ Mechanical force can serve as a trigger to activate or amplify the therapeutic properties of nanoparticles, enhancing their effectiveness in targeting disease cells. The ability to modulate the activity of L1 nanoparticles through sonication provides a versatile approach to cancer therapy, with the therapeutic effect being precisely controllable by adjusting the intensity and duration of sonication.

This study supports the growing body of evidence on the therapeutic potential of sonicated piezocatalytic nanoparticles for cancer treatment. The enhanced cytotoxicity against cancer cells, coupled with the low toxicity to normal cells, suggests that L1 nanoparticles may offer a targeted approach for cancer therapy. The underlying piezocatalytic mechanism, which involves ROS generation upon sonication, plays a crucial role in the nanoparticles' anticancer effects, while the sonication-induced enhancement of cytotoxicity adds a valuable dimension to their therapeutic potential. Further investigations into the molecular mechanisms of action and the optimization of nanoparticle formulations for clinical use are warranted to fully exploit the therapeutic benefits of sonicated L1 nanoparticles.

### 4.13. Clinical translation and future perspectives

The translation of Li_2_TiO_3_ nanoparticles into clinical applications presents both substantial opportunities and critical challenges that warrant careful consideration. The demonstrated ability to achieve potent antimicrobial effects through mechanically-activated ROS generation, coupled with favorable cytotoxicity profiles at therapeutic concentrations, establishes a promising foundation for clinical development. The optimization of delivery systems represents a primary challenge for clinical implementation, particularly concerning the maintenance of piezocatalytic properties in complex physiological environments. Studies by Yu *et al.* have highlighted the critical importance of particle size and surface characteristics in determining biodistribution patterns and therapeutic efficacy.^121^ Furthermore, variations in local mechanical forces across different physiological environments necessitate tissue-specific optimization of delivery strategies.^122^ These considerations must be carefully balanced against the need for standardized treatment protocols that can be reliably implemented in clinical settings. Long-term safety and biocompatibility considerations present additional challenges that require systematic investigation. While our findings demonstrate favorable cytotoxicity profiles at therapeutic concentrations, comprehensive evaluation of tissue-specific responses and potential accumulation effects remains crucial. The potential impact on host microbiome must also be carefully evaluated, particularly given the increasing recognition of microbiome importance in health and disease outcomes.

The mechanical activation dependency of these materials introduces unique opportunities for controlled therapeutic delivery while requiring careful consideration of activation methods in clinical settings. Recent research by Yuan *et al.* indicates that ultrasound-mediated activation offers a promising approach for clinical implementation, though standardization of activation parameters and development of tissue-specific protocols remain crucial challenges.^123^ The demonstrated memory effect, while providing advantages for sustained antimicrobial activity, necessitates careful investigation of long-term tissue responses and potential adaptive mechanisms.^41^

The development of resistance monitoring strategies represents another critical aspect of clinical translation. While the multi-modal mechanism of action may reduce the likelihood of resistance development, systematic investigation of potential bacterial adaptation mechanisms remains essential. Monitoring stress response pathways and potential cross-resistance with conventional antibiotics is crucial for developing effective resistance mitigation strategies.^124^ Additionally, the establishment of standardized protocols for evaluating therapeutic efficacy and monitoring potential resistance development will be essential for successful clinical implementation.

These findings collectively establish new paradigms for mechanically-activated antimicrobial therapy while highlighting crucial areas for future investigation. The unique combination of controlled activation, sustained therapeutic effects, and selective toxicity positions Li_2_TiO_3_ NPs as promising candidates for addressing the growing challenge of antimicrobial resistance. Future research should focus on optimizing material properties for enhanced therapeutic efficacy, developing tissue-specific activation protocols, investigating combination therapy approaches, and establishing comprehensive clinical safety protocols. The successful translation of these findings into clinical practice could provide valuable new tools for combating resistant infections while minimizing adverse effects on host tissues and beneficial microbiota.

## 5. Conclusion

This study demonstrates that hydrothermally synthesized Li_2_TiO_3_ nanoparticles can be optimized into a multifunctional piezocatalytic nanoplatform for antimicrobial and anticancer applications. Structural and physicochemical characterization confirmed the formation of phase-pure, highly crystalline monoclinic Li_2_TiO_3_ nanoparticles with a uniform diamond-shaped morphology (25–80 nm), homogeneous elemental distribution, and characteristic FTIR absorption bands. BET analysis further demonstrated that methylene blue removal was predominantly governed by piezocatalytic degradation rather than adsorption, confirming the intrinsic catalytic activity of the optimized nanoparticles. Upon ultrasonic activation, the optimized L1 formulation exhibited superior piezocatalytic performance, achieving 36.69% methylene blue degradation together with significantly enhanced ROS generation. The increased ROS production translated into potent antibacterial activity against multidrug-resistant *S. aureus* and extensively drug-resistant *P. aeruginosa*, resulting in markedly reduced MIC and MBC values and complete bacterial eradication within 48 h. In addition, the nanoparticles demonstrated exceptional antibiofilm efficacy, inhibiting biofilm formation by 99.56% and removing established biofilms by 99.95%. Mechanistic investigations further demonstrated that bacterial inactivation was mediated through ROS-induced plasma membrane depolarization and severe membrane disruption, providing direct evidence for the underlying antibacterial mechanism. The optimized nanoparticles also exhibited favorable hemocompatibility with minimal lithium-ion release, supporting their further preclinical evaluation for biomedical applications. Furthermore, they demonstrated significant cytotoxic activity against HCT-116 colorectal cancer cells, with an IC_50_ of 100 µg mL^−1^ and an LC_50_ of 200 µg mL^−1^. Flow cytometric and SEM analyses revealed increased cell death, disruption of membrane integrity, and pronounced morphological alterations following treatment. The sustained antibacterial and anticancer activities observed after sonication further indicate that ultrasonic activation not only enhances the immediate biological response but also prolongs the therapeutic efficacy of the nanoparticles.

Taken together, the favorable structural characteristics, efficient ROS-mediated piezocatalytic activity, potent antibacterial and antibiofilm efficacy, favorable biocompatibility, and significant anticancer activity demonstrate the multifunctional potential of Li_2_TiO_3_ nanoparticles for biomedical applications. These findings provide a strong scientific foundation for the continued development of Li_2_TiO_3_-based piezocatalytic nanomaterials as alternative strategies for combating antimicrobial resistance and colorectal cancer. Future studies should focus on *in vivo* evaluation, comprehensive biosafety assessment, and optimization of ultrasound-assisted therapeutic protocols to facilitate their progression toward clinical translation.

## Author contributions

Karzan Qurbani: writing – original draft, visualization, validation, software, methodology, investigation, formal analysis, data curation, conceptualization, resources. Omid Amiri: review & editing, supervision, project administration, conceptualization. Haider Hamzah: review & editing, supervision, project administration, conceptualization.

## Conflicts of interest

For all authors there are no conflicts of interest, including financial, personal, or other relationships with other individuals or organizations that might inappropriately influence or could be perceived to influence their work.

## Supplementary Material

RA-OLF-D6RA05405F-s001

## Data Availability

All data are incorporated into the article and its online supplementary information (SI). Supplementary information is available. See DOI: https://doi.org/10.1039/d6ra05405f.
