# Supplementary material for: Piezocatalytic lithium titanate nanoparticles: a dual-action strategy against multidrug-resistant pathogens and cancer
Source: RSC Adv. 2026 Jul 27. Online ahead of print. doi: 10.1039/d6ra05405f (PMC13404369; doi:10.1039/d6ra05405f)
Supplement: RA-OLF-D6RA05405F-s001 [file RA-OLF-D6RA05405F-s001.pdf]

Supplementary Information (SI) for RSC Advances.
This journal is © The Royal Society of Chemistry 2026

Supporting Information

Piezocatalytic Lithium Titanate Nanoparticles: A Dual-Action Strategy Against Multidrug-Resistant Pathogens and Cancer

Karzan Qurbani [1], Haider Hamzah [2], Omid Amiri [3]

[1] Department of Biology, College of Science, University of Raparin, Rania City, 46012, Kurdistan Region, Iraq.

[2] Department of Biology, College of Science, University of Sulaimani, Sulaymaniyah City, 46001, Kurdistan Region, Iraq.

[3] Faculty of Chemistry, Razi University, and P. O. Box: 6714414971, Kermanshah, Iran.

S1. Detailed Piezocatalytic Evaluation Procedures

General conditions.

All piezocatalytic experiments were performed in the dark at 25 °C (water bath controlled) to exclude photolysis and thermocatalysis. Nanoparticle suspensions were freshly prepared before each experiment.

Methylene blue (MB) assay.

Li_2TiO_3 nanoparticles (100 μg/mL) were dispersed in 5 mL MB solution (10 μg/mL) and ultrasonicated at 300 W for 10 min using a titanium probe sonicator. Following centrifugation at 14,000 rpm for 5 min in 2 mL Eppendorf tubes, the supernatant was analyzed by UV–Vis spectroscopy at 664 nm. MB degradation served as an indirect measure of ROS generation (modified from [111].

DPBF assay for $^1O_2$ and $•O_2^-$.

A 1 mL nanoparticle suspension (25 μg/mL) was mixed with 20 μL DPBF solution (1 mg/mL in ethanol). After 10 min ultrasonication (300 W), the decrease in absorbance at 410 nm was monitored by UV–Vis.

Terephthalic acid assay for •OH.

Terephthalic acid (0.25 mg) was dissolved in 3 mL NaOH (2 mM) and combined with 3 mL nanoparticle suspension (200 μg/mL). The mixture was ultrasonicated (300 W, 10 min), and fluorescence spectra were collected at Ex 315 nm/Em 425 nm (Hitachi F-7000, Japan), modified from [40].

S2. MB Degradation Screening and Optimization

Formulation screening (L1–L9).

Each formulation (100 μg/mL) was dispersed in MB solution (10 μg/mL, 5 mL) and ultrasonicated at 300 W for 10 min. Absorbance at 664 nm before and after treatment was used to calculate degradation efficiency. The highest performing formulation (L1) was selected for optimization.

Sonication time optimization.

Aliquots of MB solution (10 μg/mL) containing L1 nanoparticles (100 μg/mL) were subjected to ultrasound irradiation (300 W) for 0, 1, 4, 7, 10, 15, 30, and 60 min. A reference control of MB solution (10 μg/mL) without nanoparticles was included under identical conditions. Degradation efficiency was quantified by UV–Vis spectrophotometry at 664 nm.

Ultrasonication power optimization.

L1 suspensions (100 µg/mL) were sonicated for 10 min at different power levels (0, 100, 200, 300, 400, 500 W). MB degradation efficiency was measured at 664 nm.

On–off ultrasonication cycles.

L1 suspensions (100 µg/mL) in MB solution (10 µg/mL) were subjected to four cycle patterns: continuous, 1–3 s, 2–2 s, and 3–1 s, with total sonication time fixed at 10 min. Degradation efficiency was quantified by UV–Vis at 664 nm.

Evaluation of Memory Effect of Piezocatalytic Activity

To assess the memory effect of the piezocatalytic activity, the degradation of MB was measured at various time intervals following the initial sonication. After the 10-minute sonication, the reaction mixture was left undisturbed, and absorbance at 664 nm was recorded at 24, 48, 72, and 96 hours. The goal of this assessment was to evaluate how long the Li_2TiO_3 nanoparticles retained their piezocatalytic activity without additional sonication, providing insights into their long-term stability and potential for sustained catalytic performance.

Table S1. Synthesis parameters for Li_2TiO_3 nanoparticles under varying autoclave temperature, duration, precursor concentration, and autoclave solution volume.

Code	Autoclave time	Autoclave temperature	Concentration	Volume
L1	24 hours	180 °C	0.08 mol (3.3568 g) LiOH + 0.04 mol (3.1912 g) TiO2 in 40 ml DW	70%
L2	20 hours	180 °C	0.08 mol (3.3568 g) LiOH + 0.04 mol (3.1912 g) TiO2 in 40 ml	70%
L3	16 hours	180 °C	0.08 mol (3.3568 g) LiOH + 0.04 mol (3.1912 g) TiO2 in 40 ml	70%
L4	24 hours	210 °C	0.08 mol (3.3568 g) LiOH + 0.04 mol (3.1912 g) TiO2 in 40 ml	70%
L5	24 hours	150 °C	0.08 mol (3.3568 g) LiOH + 0.04 mol (3.1912 g) TiO2 in 40 ml	70%
L6	24 hours	180 °C	0.054 mol (2.2658 g) LiOH + 0.028 mol (2.2338 g) TiO2 in 40 ml	70%
L7	24 hours	180 °C	0.104 mol (43638 g) LiOH + 0.054 mol (4.308 g) TiO2 in 40 ml	70%
L8	24 hours	180 °C	0.08 mol (2.4 g) LiOH + 0.04 mol (2.28 g) TiO2 in 28.6 ml	50%
L9	24 hours	180 °C	0.08 mol (4.3218 g) LiOH + 0.04 mol (4.109 g) TiO2 in 51.5 ml	90%

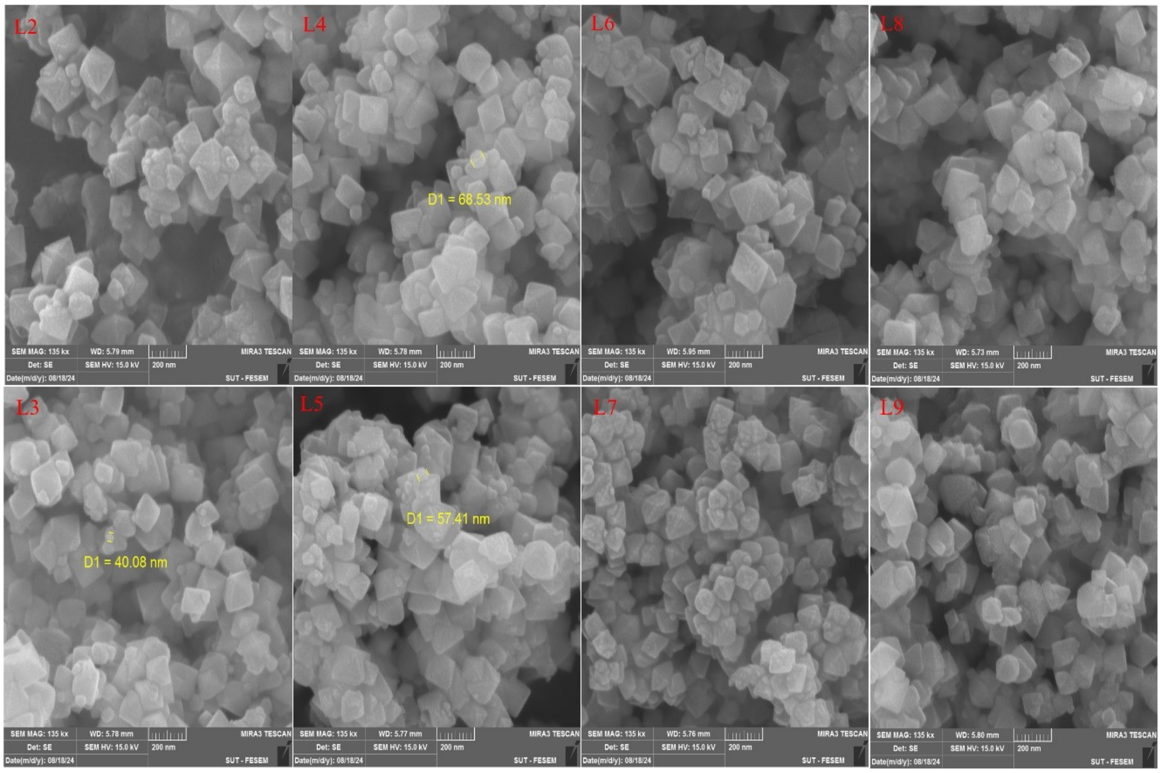

Supplementary Figure S1. SEM micrographs of L2 to L9.

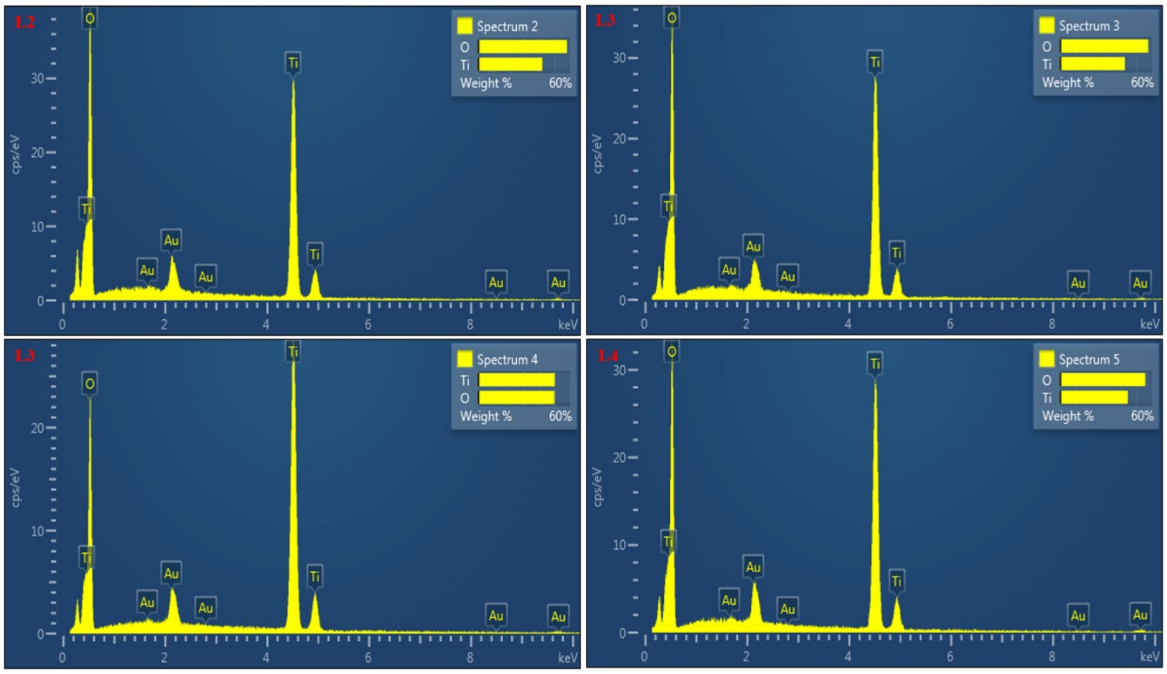

Supplementary Figure S2. EDX spectra of L2 to L5, detailing the elemental composition of the synthesized materials.

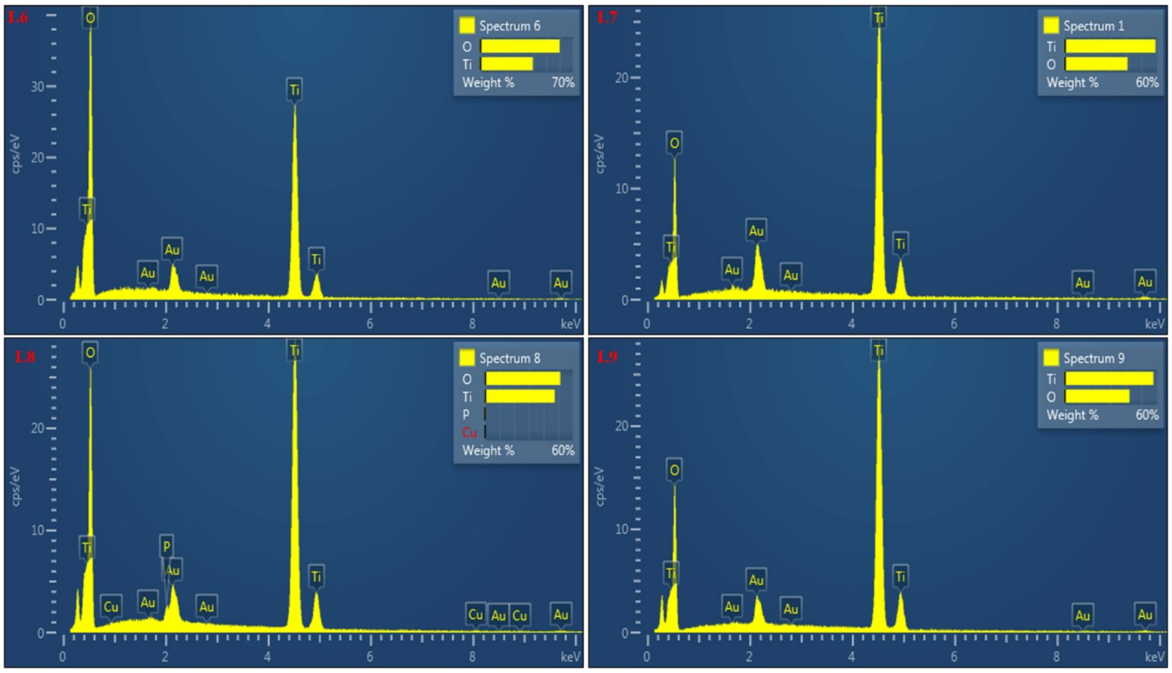

Supplementary Figure S3. EDX spectra of L6 to L9, detailing the elemental composition of the synthesized materials.

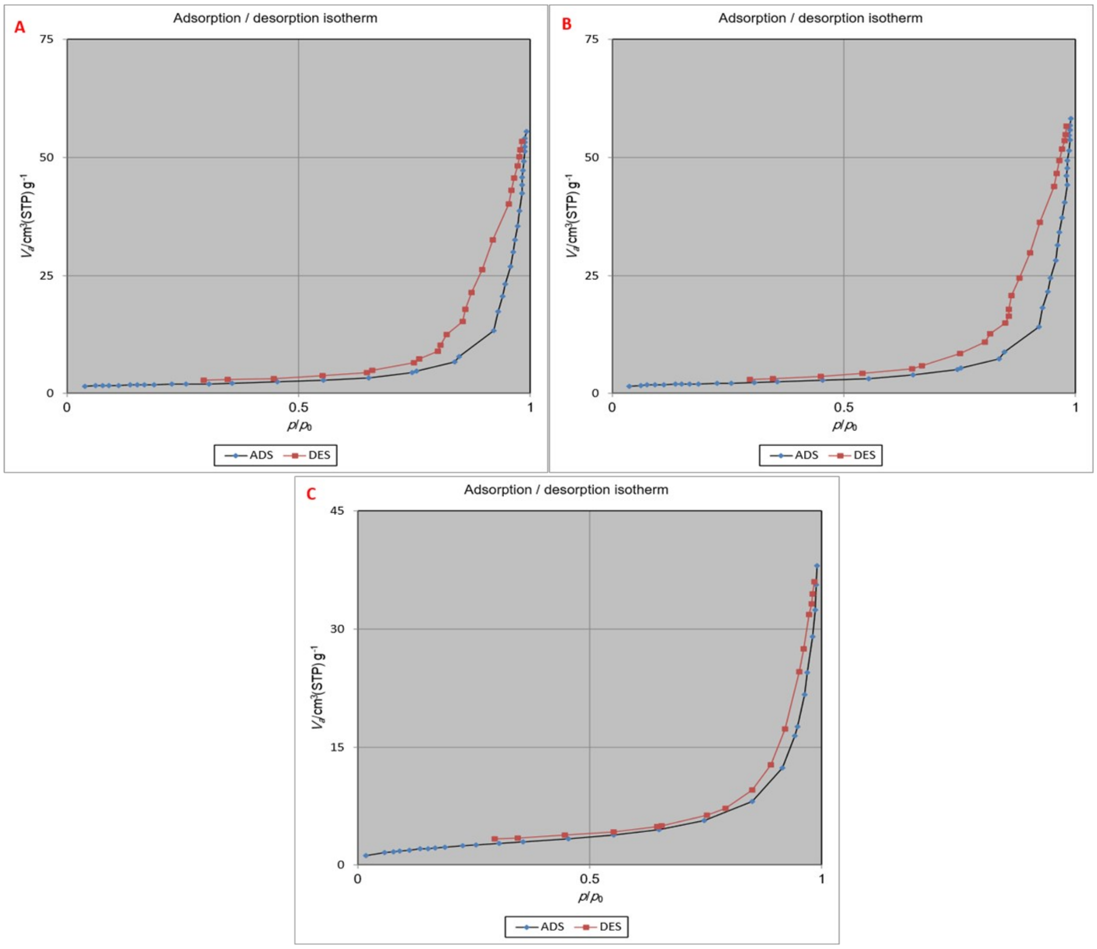

Supplementary Figure S4. BET surface area analysis of samples. (A) L2, (B) L3, (C) L7.

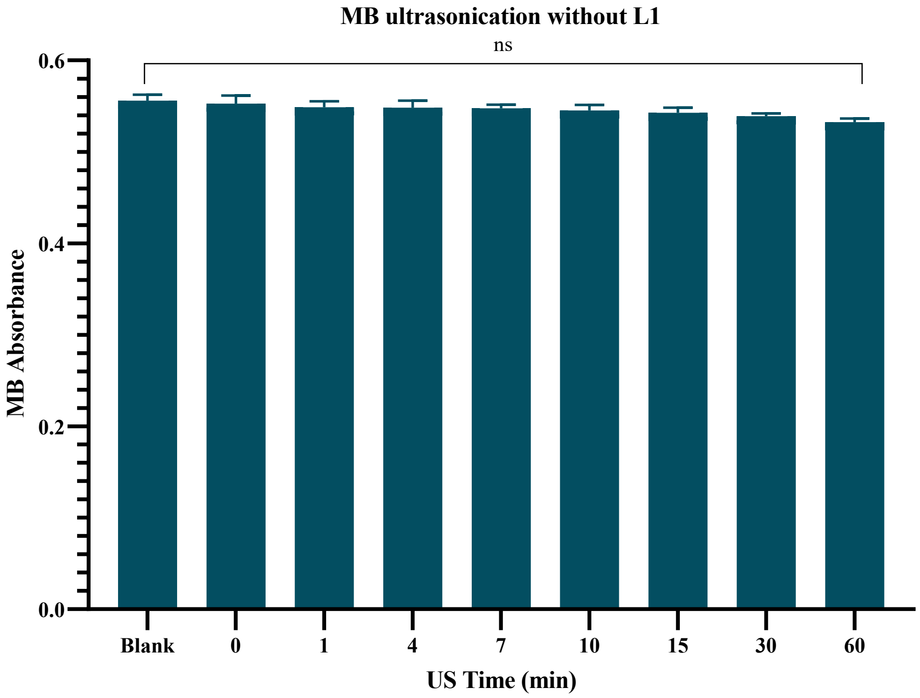

Figure S5. MB degradation under ultrasound (300 W, 1–60 min) without Li_2TiO_3 nanoparticles, showing limited discoloration compared to nanoparticle-mediated piezocatalysis.

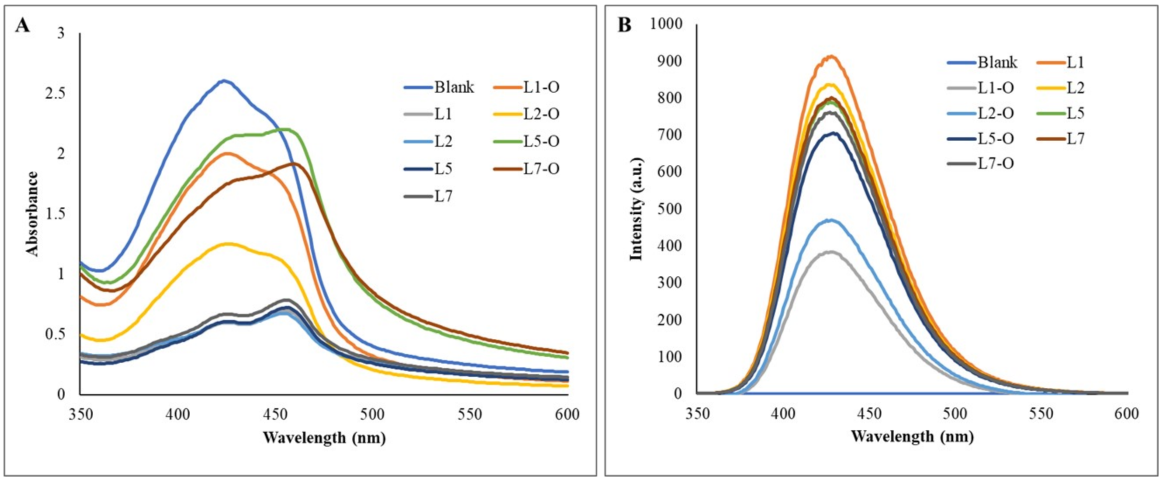

Figure S6. ROS Generation in Li_2TiO_3 NPs. The figure illustrates the generation of reactive oxygen species (ROS) in different Li_2TiO_3 nanoparticle formulations, including sonicated (L1) and non-sonicated (L1-O) versions, as well as L2, L2-O, L5, L5-O, L7, and L7-O. (A) Singlet oxygen ($^1O_2$) and superoxide anion ($\bullet O_2^-$), (B) Hydroxyl radicals ($\bullet OH$).

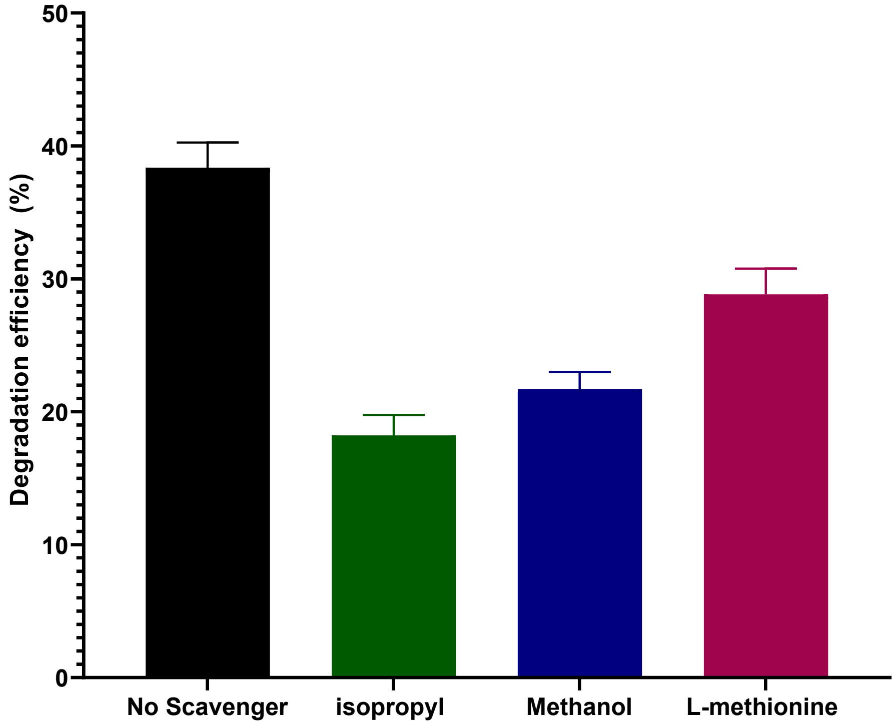

Figure S7. Scavenger-trapping analysis of MB degradation by L1 nanoparticles

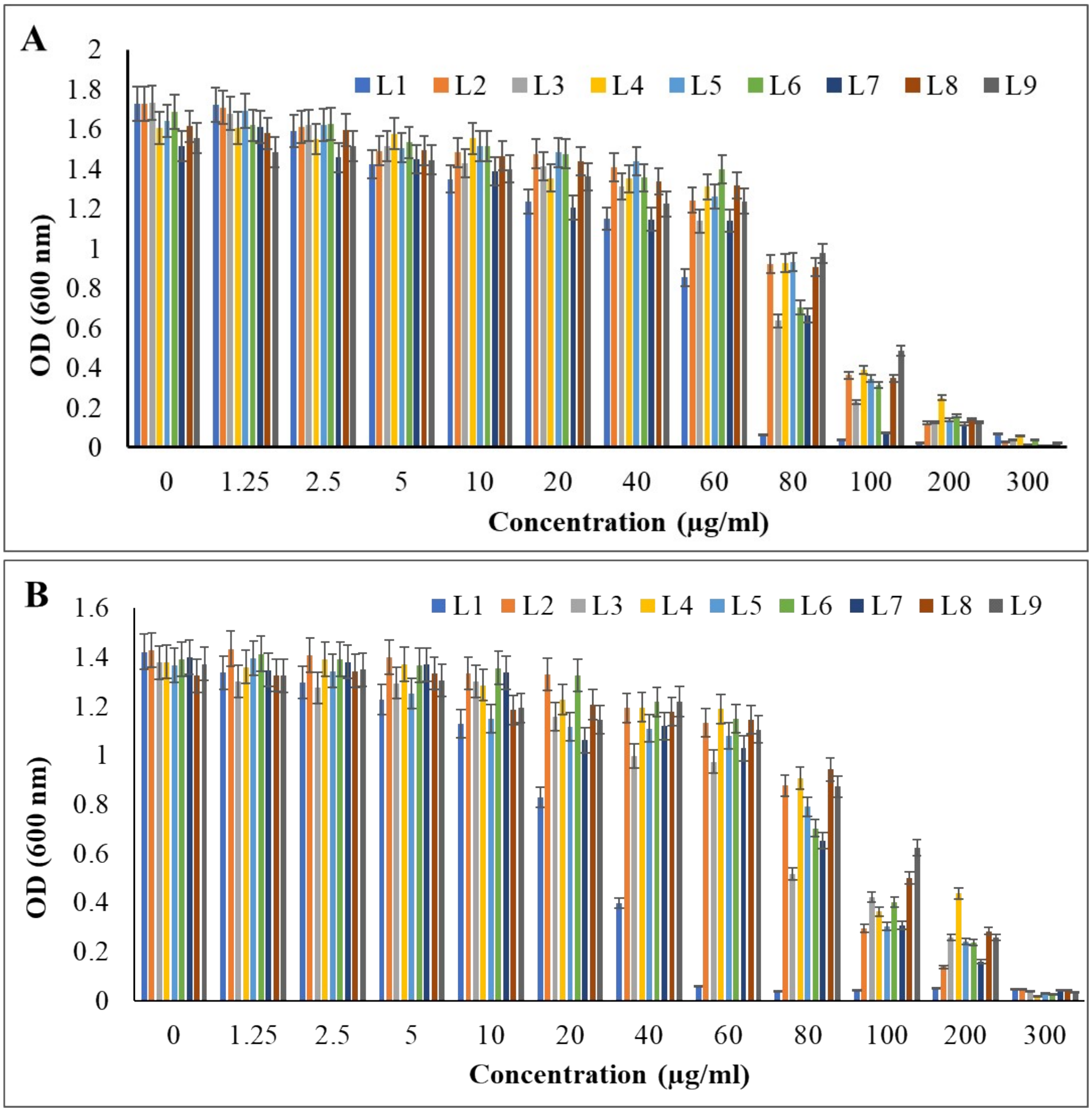

Figure S8. Antibacterial activity of Li_2TiO_3 nanoparticles (L1-L9) against clinical isolates A. *S. aureus* MDR, B. *P. aeruginosa* XDR.

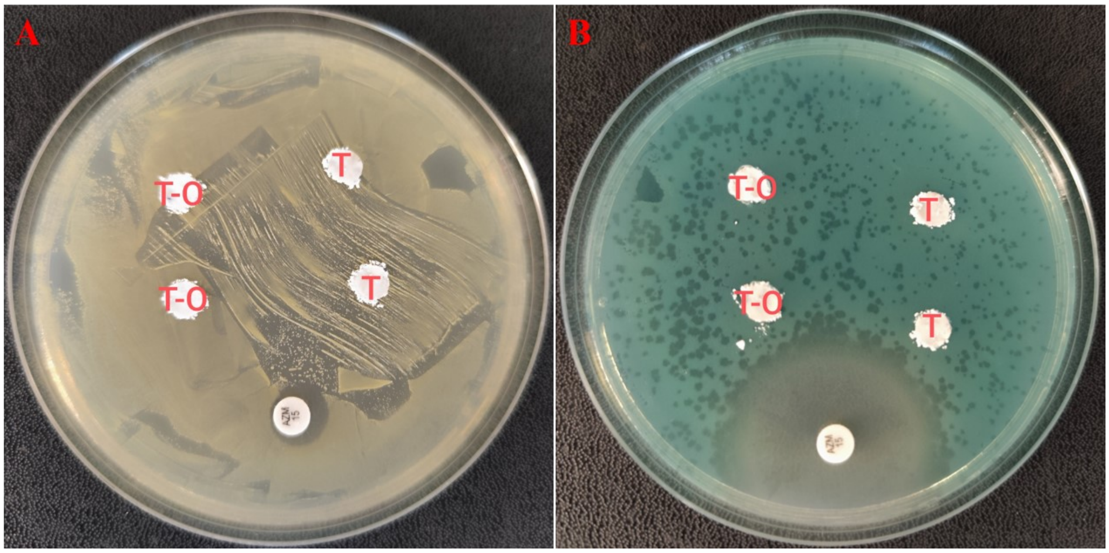

Figure S9. Representative Kirby–Bauer disc diffusion assay of non-sonicated TiO_2 (T-O) and sonicated TiO_2 (T) against (A) MDR *S. aureus* and (B) XDR *P. aeruginosa.*

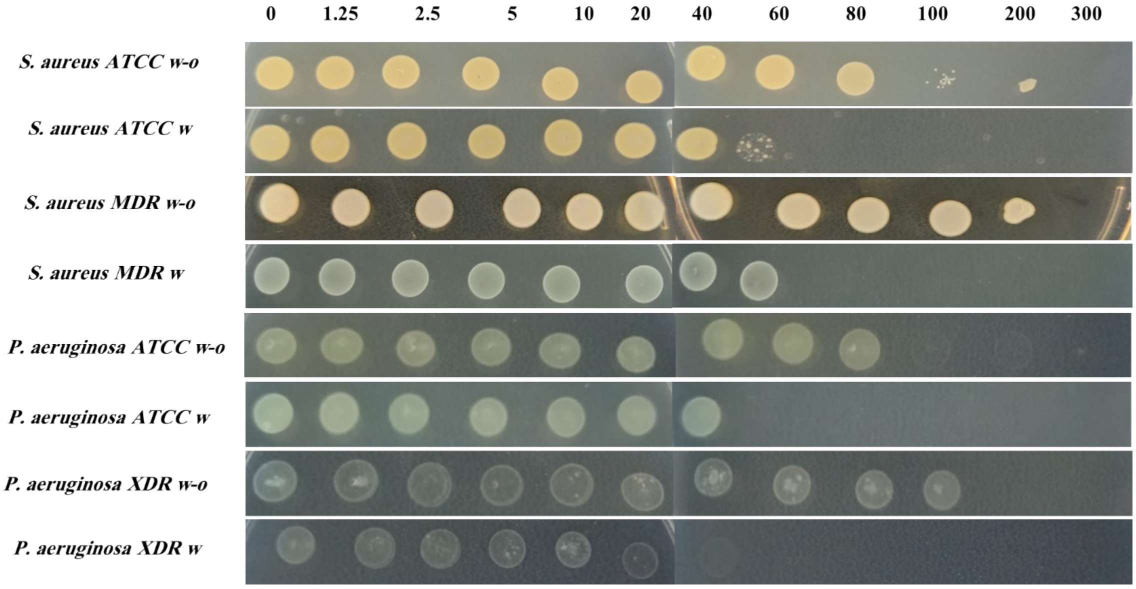

Supplementary Figure S10. Spot Assay Evaluation of Bactericidal Activity of Li_2TiO_3 NPs Against *S. aureus* (ATCC strain and MDR clinical isolate) and *P. aeruginosa* (ATCC strain and MDR clinical isolate). The assay was conducted using non-sonicated (w-o) and sonicated (w) Li_2TiO_3 NPs to confirm MBC concentrations

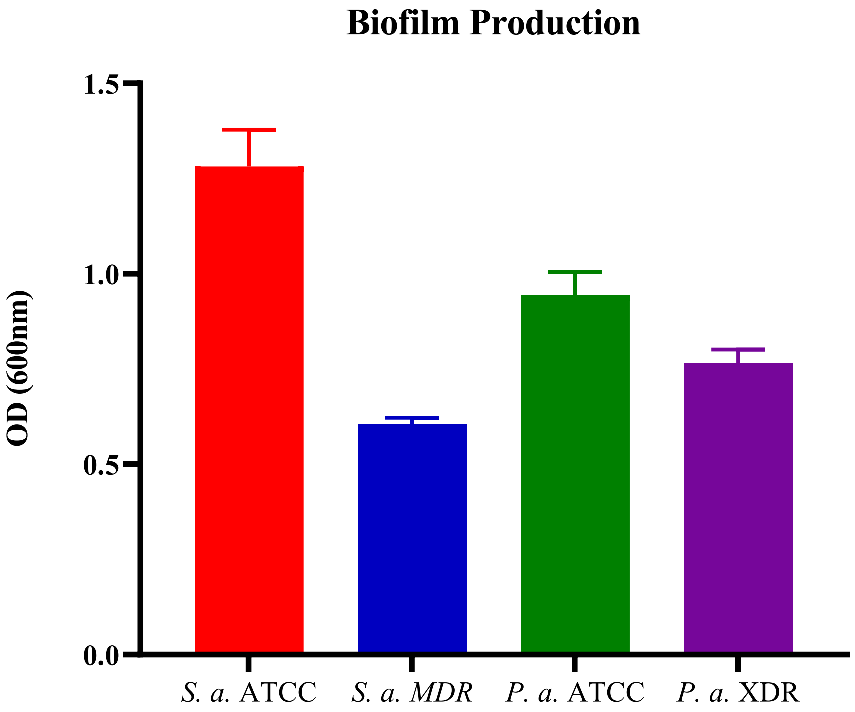

Figure S11. Biofilm production of *S. aureus* ATCC, *S. aureus* MDR, *P. aeruginosa* ATCC, and *P. aeruginosa* XDR

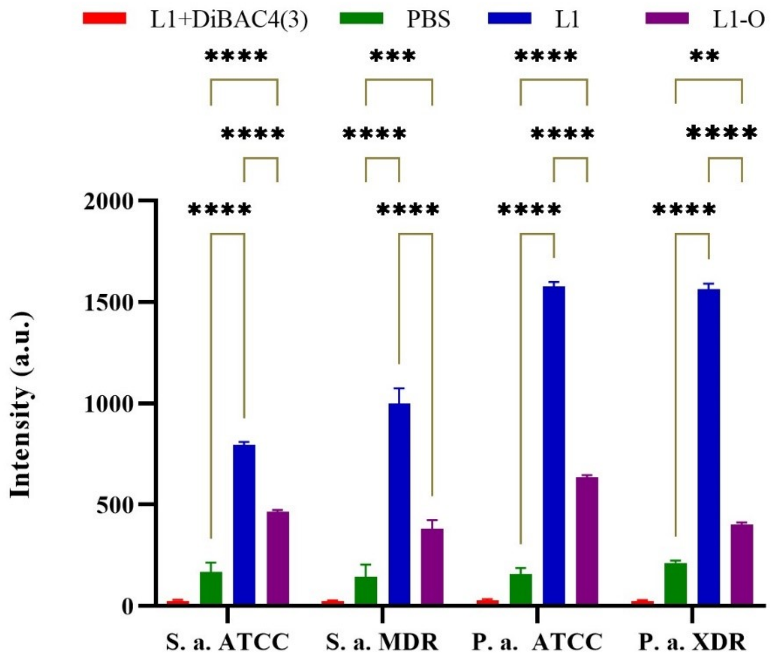

Figure S12. Plasma membrane permeability of *S. aureus* (ATCC strain and MDR clinical isolate) and *P. aeruginosa* (ATCC strain and XDR clinical isolate) strains treated with PBS (control), non-sonicated Li_2TiO_3 nanoparticles (L1-O), and sonicated Li_2TiO_3

nanoparticles (L1). Fluorescence intensity, measured using the $DiBAC_4(3)$ assay, reflects membrane permeability and damage. The results demonstrate a significant increase in permeability upon treatment with sonicated nanoparticles (L1), particularly in *P. aeruginosa* strains, compared to non-sonicated nanoparticles and the PBS control. Statistical analysis was performed using two-way ANOVA, with a significance level of $p < 0.0001$. Error bars represent the standard deviation of three independent experiments.

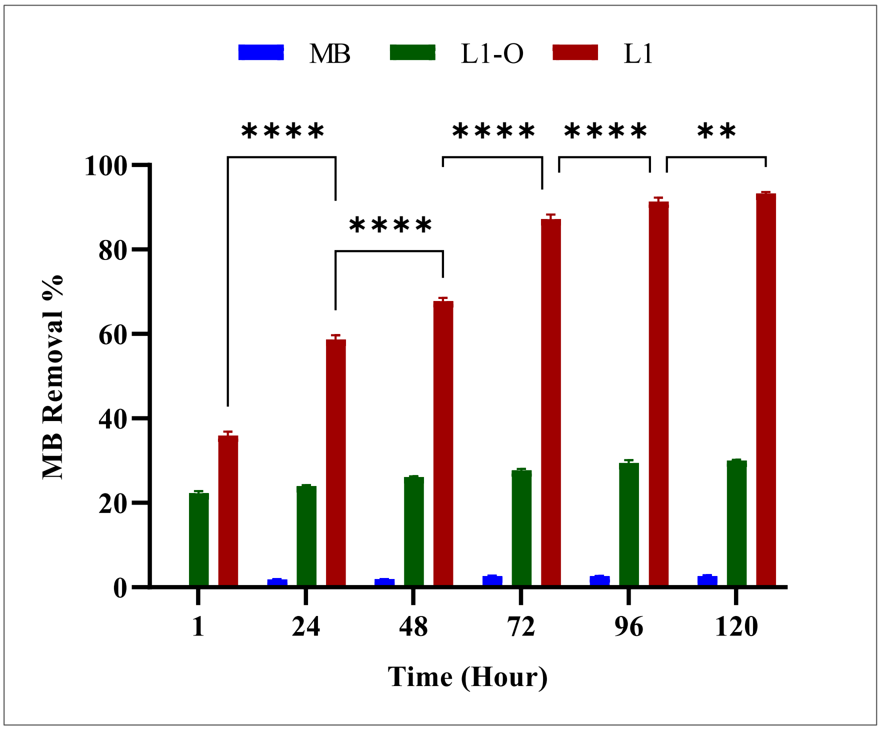

Figure S13. Memory effect of piezocatalytic activity in L1 nanoparticles
